# Clearance of Stress-Induced Premature Senescent Cells Alleviates the Formation of Abdominal Aortic Aneurysms

**DOI:** 10.14336/AD.2023.0215

**Published:** 2023-10-01

**Authors:** Jingfang Xie, Zhenquan Tang, Qiqi Chen, Xiaoqian Jia, Chuling Li, Ming Jin, Guoquan Wei, Hao Zheng, Xinzhong Li, Yanmei Chen, Wangjun Liao, Yulin Liao, Jianping Bin, Senlin Huang

**Affiliations:** ^1^Department of Cardiology, State Key Laboratory of Organ Failure Research, Nanfang Hospital, Southern Medical University, Guangzhou, China.; ^2^Guangdong Provincial Key Laboratory of Cardiac Function and Microcirculation, Guangzhou, China.; ^3^Department of Oncology, Nanfang Hospital, Southern Medical University, Guangzhou, China.

**Keywords:** stress-induced premature senescence, abdominal aortic aneurysm, ABT263, phenotypic switch, FGF9

## Abstract

Abdominal aortic aneurysm (AAA) is a multifactorial disease characterized by various pathophysiological processes, including chronic inflammation, oxidative stress, and proteolytic activity in the aortic wall. Stress-induced premature senescence (SIPS) has been implicated in regulating these pathophysiological processes, but whether SIPS contributes to AAA formation remains unknown. Here, we detected SIPS in AAA from patients and young mice. The senolytic agent ABT263 prevented AAA development by inhibiting SIPS. Additionally, SIPS promoted the transformation of vascular smooth muscle cells (VSMCs) from a contractile phenotype to a synthetic phenotype, whereas inhibition of SIPS by the senolytic drug ABT263 suppressed VSMC phenotypic switching. RNA sequencing and single-cell RNA sequencing analysis revealed that fibroblast growth factor 9 (FGF9), secreted by stress-induced premature senescent VSMCs, was a key regulator of VSMC phenotypic switching and that FGF9 knockdown abolished this effect. We further showed that the FGF9 level was critical for the activation of PDGFRβ/ERK1/2 signaling, facilitating VSMC phenotypic change. Taken together, our findings demonstrated that SIPS is critical for VSMC phenotypic switching through the activation of FGF9/PDGFRβ/ERK1/2 signaling, promoting AAA development and progression. Thus, targeting SIPS with the senolytic agent ABT263 may be a valuable therapeutic strategy for the prevention or treatment of AAA.

## INTRODUCTION

Abdominal aortic aneurysm (AAA), characterized as a regional dilation of the aortic diameter of >50%, is one of the major causes of mortality worldwide [1]. The only treatment for AAA is surgical repair for patients with symptoms or > 55 mm in maximum diameter, while early elective surgical repair of smaller AAAs (<55 mm) is not beneficial [2]. As over 95% of AAAs identified were <55 mm in diameter[3], the development of a noninvasive strategy to limit or prevent the progressive expansion and rupture of the aneurysm is awaited. Previous research has demonstrated that medications targeting risk factors for AAA, including hypertension and dyslipidemia, have the potential to limit AAA progression [2]. Unfortunately, the data from clinical trials showed that intervention in risk factors for AAA achieved only moderate or even negative effects on slowing AAA progression [4]. The limited success in clinical practice is mainly due to the complex [5], multifactorial and even confusing [2] etiology of AAA, which might be difficult to effectively block this condition. Moreover, the pathophysiological process initiated by risk factors for AAA has a durable influence on AAA formation [6]. Intervention in risk factors alone was insufficient to block the continuity of the initiated pathogenic process for AAA. Targeting the pathogenic process that mediates the effect of risk factors on initiating vascular remodeling of AAA was suggested to have greater accuracy and efficiency [7], serving as a potent therapeutic approach. Research has focused on experimental therapies based on the pathophysiological process of the disease [1] initiated by risk factors. The pathological features of AAA include chronic aortic inflammation, extracellular matrix (ECM) degradation, and depletion of vascular smooth muscle cells (VSMCs) [8]. Recent studies have revealed that multiple agents, including anti-inflammatory agents and inhibitors of MMPs, directly impede or halt pathological vascular remodeling in animals and alleviate AAA progression to a certain extent [9]. Thus, a pharmacological solution that blocks the bridge linking risk factors and these pathophysiological processes might be an attractive alternative strategy for AAA prevention and treatment.

Chronic exposure to risk factors for vascular diseases, such as hypertension [10], nicotine [11], and hyperlipidemia [12], promotes premature senescence in vascular cells by generating stress-dependent damage [13]. Stress-induced premature senescence (SIPS) has been found to be one of the main contributors to the formation of vascular diseases, including aortic atherosclerosis [14] and vascular calcification [15], indicating its relationship to AAA. More importantly, an accumulating body of evidence has indicated that SIPS might be essential for mediating the effect of risk factors on initiating the pathogenesis of AAA. First, SIPS is closely associated with the inflammatory response in injured tissues, such as the heart [16], kidney [17], lung [18] and liver [19]. Previous studies confirmed that stress-induced premature senescent cells could secrete a cocktail of pro-inflammatory cytokines and chemokines, recruiting a large number of inflammatory cells into the vascular wall [20]. In addition, compared to normal cells, premature senescent cells have reduced expression of antioxidant enzymes [21]. Accordingly, SIPS could lead to excessive production of reactive oxygen species (ROS) [22], thereby increasing oxidative stress-related damage in the aorta. Furthermore, stress-induced premature senescent cells induce the degradation of the ECM by the secretion of matrix proteases [23], which contributes to destruction of the aortic middle layer and the subsequent dilation of the aorta [24]. The link between risk factors for AAA and SIPS, as well as the integrative effect of SIPS on inflammation, oxidative stress, and ECM degradation, suggested that SIPS might be essential for mediating the effect of risk factors on the pathogenesis of AAA. Therefore, the development of interventions for SIPS might efficiently suppress AAA formation or progression.

Senolytic therapy is a novel and effective pharmacological method to selectively eliminate senescent cells by targeting abnormally activated antiapoptotic pathways without affecting normal cells [25]. Previous studies revealed that senolytics could release senescent cells burden in many organs, improve physical function, and even expand the lifespan [25]. Moreover, senolytic agents were found to target senescent cells and improved functional recovery after acute injury of several tissues in aged mouse models [26]. Experiments inducing cellular senescence in young mice showed similar results after acute injury [27]. Growing evidence suggests that senolytics might be a potential pharmacological method for AAA by restraining the detrimental effect of SIPS. Senolytics could remove stress-induced premature senescent cells to decrease the secretion of senescence-associated secretory phenotype (SASP) factors [28]. A reduced SASP in the aorta contributes to the repression of inflammation and ECM degradation in the aorta, which are important to inhibit AAA progression [29]. Additionally, senolytic treatment could reverse the adverse hemodynamic changes in blood vessels [30], which could help prevent AAA rupture [2]. The above results indicated the potent role of senolytic drugs in alleviating the structural and functional changes induced by SIPS in the vasculature during AAA development. We thus hypothesized that the application of senolytic drugs might block the pathogenesis of AAA by inhibiting SIPS.

In the current study, we aimed to determine the existence of SIPS in human and mouse AAA samples and explore whether inhibition of SIPS by senolytics could prevent or slow AAA progression. Moreover, we sought to investigate the underlying mechanism by which SIPS affects AAA pathology. Our findings indicated that targeting SIPS with senolytic agents might be a powerful therapeutic approach to postpone AAA progression.

## MATERIALS AND METHODS

### Human Aortic Samples

Human aortic samples were obtained according to a multicenter clinical research project approved by the Ethics Committees of NanFang Hospital (ethical approval number: NFEC-2019-086). All procedures complied with the principles of the Declaration of Helsinki. Human tissue samples were collected from the abdominal aorta of patients undergoing open surgical repair. Adjacent nonaneurysmal aortic sections were collected from the same patients and served as a control group. In addition, this multicenter clinical research project received an informed consent exemption from the ethics committee of the NanFang Hospital. The removed aortic tissue was quickly flash-frozen in liquid nitrogen and stored at -80 °C for subsequent treatment. Patient clinical information is available in Supplementary Table 1.

### Experimental Animals

All animal procedures were approved by the Institutional Animal Care and Use Committee at Southern Medical University. Nine- to twelve-week-old male C57BL/6J mice and apolipoprotein E-deficient (ApoE^-/-^) mice were purchased from Southern Medical University. All mice were raised under pathogen-free conditions with a 12-h dark/12-h light cycle and a humidity of 60%-65%. The care and experimental procedures of the animals were in accordance with the NIH Animal Research Advisory Committee Guidelines.

### Angiotensin II (Ang II) Infusion Model

The Ang II-induced AAA model was established as previously described [31]. The mice were anesthetized with 2% isoflurane. An osmotic minipump (Alzet, Model 2004, Durect Corporation, Cupertino, CA, USA) equipped with Ang II (1000 ng/kg/min, A9525, Sigma, St. Louis, MO, USA) or normal saline was subcutaneously implanted through a small incision in the dorsum of the neck. After 28 days of infusion, the mice were euthanized, and the aortas were harvested for further assessment.

### Elastase-Induced AAA Model

The mice were fully anesthetized before being subjected to laparotomy. The abdominal aortic segment from the renal artery to the iliac artery was separated from the surrounding retroperitoneal structures. A cotton gauze soaked with elastase solution (E1250, Sigma-Aldrich) or NaCl (0.9%) was applied to the abdominal aorta of the mice for 15 min. After 2 weeks, the mice were sacrificed, and the elastase-treated aortic segment was harvested for morphological and histological analyses.

### In Vivo ABT263 (Navitoclax) Treatment

ABT263 was prepared in a lipid vehicle solution consisting of EtOH, polyethylene glycol 400 and Phosal 50 PG in a 1:3:6 ratio [27]. Mice were randomly divided into different experimental groups. The dose-timing regimen of ABT263 (50 mg/kg/day, oral gavage; A3007, Apexbio) is described in detail in the text. The dosage and mode of administration of ABT263 were determined according to previous studies [27, 32].

### VSMC Isolation and Culture

Primary VSMCs were prepared from adult C57BL/6J mouse aortas as described previously [31]. Primary VSMCs were cultured with Dulbecco's modified Eagle’s medium (DMEM; Sigma-Aldrich) containing 10% fetal bovine serum (FBS; Gibco) and 100 µg/mL penicillin and streptomycin (P/S, Thermo Fisher Scientific). Cell cultures were maintained at 37 °C in a humidified 5% CO_2_ atmosphere. Cells at passages 4-6 were used for the experiments.

### Ultrasonography for AAA

Mice were anesthetized with 2% isoflurane and then subjected to ultrasound one day before angiotensin infusion as a baseline and on the 14th and 28th days. Ultrasonic B-mode images of abdominal aortas were obtained using a Vevo 2100 imaging system (Visual Sonics, ON, Canada) equipped with a 40-MHz probe.

### Aneurysm Quantification

For identification of aortic aneurysms, the mice were euthanized, and the abdominal cavity was opened by a midventral incision. Mice underwent whole-body perfusion-fixation via the left ventricle with paraformaldehyde at physiological pressure. Then, the aortas were isolated and photographed using a digital camera. The maximum outer width of the dilated portion of the suprarenal aorta for the Ang II-induced model and the infrarenal aorta for the PPE-induced model was measured using Image-Pro Plus software (Media Cybernetics, USA). The mice were necropsied if they died during the experiment. The ruptured aortas were used only to calculate mortality and were not included in the maximal aortic diameter analysis. Aneurysm occurrence was confirmed as an increase in maximal aortic diameter by at least 50% compared with that of the controls. AAA evaluation was performed by two investigators who were completely blinded to the group information.

### Histological Analyses

Mice were euthanized, and the exposed aorta was injected with saline to remove blood. The aorta was perfused with paraformaldehyde for 5 min under physiological stress. Suprarenal abdominal aortas (for Ang II-induced AAA models) or infrarenal abdominal aortas (for elastase-induced AAA models) were harvested and fixed in paraformaldehyde for 24 hours. Then, aortic samples were embedded in paraffin. Serial sections (5 μm each) were obtained at intervals of approximately 500 μm. At least 10 sections were observed in each animal. Paraffin sections were then further used for H&E staining, elastin van Gieson staining, Masson’s trichrome staining and immunostaining.

### RNA Extraction and Quantitative Real-Time Polymerase Chain Reaction (qPCR)

Total RNA from abdominal aortic tissues or cultured cells was extracted using TRIzol reagent (15596026, Invitrogen) after homogenization according to the manufacturer's protocol. Total RNA was then converted to cDNA by PrimeScript™ RT Master Mix (TaKaRa Biotechnology, Dalian, China). Real-time PCR was performed with Light Cycler 480 II equipment (Roche Diagnostics, Basel, Switzerland) using a SYBR Green RT-PCR Kit (TaKaRa Biotechnology). Glyceraldehyde-3-phosphate dehydrogenase (GAPDH) and β-actin (ACTB) mRNA expression was detected as internal references to normalize the relative gene expression using the 2-ΔΔCt method. The primers used were synthesized by Tsingke (Guangzhou, China), and the primer sequences are available in Supplementary Table 2.

### Immunohistochemistry Analysis

Paraffin sections were routinely deparaffinized and rehydrated for further antigen retrieval according to the antibody manufacturer’s instructions. Endogenous peroxidase was blocked with 3% hydrogen peroxide (H_2_ O_2_ ), followed by incubation with 5% normal goat serum (diluted with PBS) at room temperature for 30 min to block nonspecific binding sites. Next, slices were incubated with primary antibodies at 4 °C overnight. Horseradish peroxidase-conjugated secondary antibody was subsequently used to incubate slices at room temperature for 30 minutes. The sections were then stained with diaminobenzidine and counterstained with hematoxylin. Finally, the slices were rinsed, dehydrated, and sealed with neutral resins. Primary antibodies were replaced with normal mouse or rabbit serum at the same concentration for immunostaining controls. Our results showed that all samples of immunostaining controls were immunonegative. The primary antibodies used were p16 (1:200; ab189034, Abcam) p21 (1:1000; ab188224, Abcam), p21 (ZM-0206, ZSGB-BIO), αSMA (1:10000; ab7817, Abcam), SM22α (1:100; 10493-1-AP, Proteintech), and Bcl2 (1:100; K003505P, Solarbio).

### Immunofluorescence Analysis

After fixation in 4% polyformaldehyde for 30 min, the aortic tissue sections or cultured cells were washed and permeabilized with 1% Triton X-100 for 5 min and blocked with 3% bovine serum albumin (BSA) for 1 h at room temperature. Then, aortic tissue sections or cultured cells were incubated with primary antibodies against p16, p21, α-SMA and SM22α overnight at 4 °C, followed by incubation for 1 hour at room temperature with fluorescent secondary antibodies (Alexa Fluor 488 and/or 594; Abcam). Next, 4',6-diamidino-2-phenylindole (DAPI; ab104139; Abcam) was used for nuclear staining, and final images were captured by confocal laser-scanning microscopy (Leica). The primary antibodies used were p16 (1:50; ab189034, Abcam), p21 (1; 10355-1-AP, Proteintech), SM22α (1:100; 10493-1-AP, Proteintech) and αSMA (1:1000; ab7817, Abcam).

### Elastin Staining and Degradation

Victoria blue van Gieson (VVG) staining was performed by applying a commercial kit (GenMed, Qingdao) according to the manufacturer's protocol. Serial sections (5 μm each) were created at intervals of approximately 500 μm. At least 10 sections were observed in each animal. Elastin degradation was evaluated by counting the number of breaks per aorta using ×40 magnification. The elastin degradation score was assessed following the previously established criteria [33]: score 1 indicates no elastin degradation; score 2 means mild elastin degradation with interruptions or breaks in the lamina; score 3 indicates moderate elastin degradation with numerous interruptions or breaks in the lamina; and score 4 indicates severe elastin degradation with fragmentation or loss or aortic rupture. The measurements were performed at least three times by two coworkers blinded to the group information.

### Masson’s Trichrome Staining

Paraffin sections were deparaffinized and then used for Masson’s trichrome staining (MST 8004; MST Biotechnology) according to the manufacturer’s instructions. The percentage of collagen area in the aortas was measured by detecting collagen deposition (blue) using ImageJ software (National Institutes of Health, Bethesda, MD).

### DHE Staining

To evaluate ROS production in aortic tissues from patients, the abdominal aortas were snap-frozen and embedded in optimal cutting temperature compound for subsequent dihydroethidium (DHE) staining. The sections (10 μm) were incubated with DHE (Molecular Probes, USA) at 37 °C for 30 min. ROS content (red fluorescence) was detected with confocal microscopy.

### In Vitro Cellular Premature Senescence Induction and Treatment

Primary mouse aortic VSMCs were used when they were grown to 70% confluence before treatment with different agents. The cells were then serum-starved and treated with Ang II at a pathological concentration (10^-7^ mol/L) for 3 days to induce SIPS in vitro according to the previous studies [34]. Next, cells were administered ABT263 at different concentrations for another 24 hours in additional experiments. For further coculture experiments, Ang II-induced premature senescent VSMCs with or without ABT263 intervention were plated on an insert membrane, and fresh medium was replaced after treatment. The cells were then cocultured with normal VSMCs plated on the well surface to establish a coculture system for another 36-48 hours.

### Senescence-Associated β-Galactosidase (SA-β-gal) Assay

SIPS of abdominal aortic tissues or cultured cells were determined using a SA-β-gal assay kit according to the manufacturer's protocol (C0602, Beyotime). After three washes with PBS, tissues or cells were fixed in formalin for 15 minutes and then washed and stained with SA-β-gal solution at 37 °C overnight (without CO_2_ ). Tissues or cells were finally washed with PBS and photographed.

### RNA-Seq Library Preparation and Data Analysis

To recapitulate possible changes in the transcriptome profile caused by ABT263, we subjected the suprarenal aortas from ApoE^-/-^ mice, Ang II-infused ApoE^-/-^ mice and Ang II-infused ApoE^-/-^ mice administered ABT263 to RNA isolation. Total RNA was extracted using TRIzol reagent according to the manufacturer’s protocol. After analysis of RNA purity, quantification and integrity, libraries were constructed using the TruSeq Stranded mRNA LT Sample Prep Kit (Illumina, San Diego, CA, USA) following the manufacturer’s protocol. The libraries were sequenced on an Illumina NovaSeq 6000 platform, and 150 bp paired-end reads were generated. Furthermore, differential expression analysis was performed using the DESeq (2012) R package. A P value < 0.05 and fold change > 2 or fold change < 0.5 were set as the thresholds for significantly differential expression. The resulting differentially expressed genes were used for enrichment analysis using R based on the hypergeometric distribution.

### Single-Cell RNA-Sequencing Data Processing

We obtained an AAA single-cell RNA-sequencing dataset (GSE186865) [35] from GEO (www.ncbi.nlm.nih.gov/geo). We utilized the R package to annotate our single-cell RNA-seq data. For single-cell RNA-seq analysis, a series of quality filters were applied to remove those cells that were in accord with any one of the following categories: too few genes expressed, too many genes expressed, too many UMIs, and too high mitochondrial gene expression. Then, the data were normalized and scaled, and principal component analysis (PCA) was used for dimensional reduction. The cells were then clustered and assigned to different cell types according to the expression of marker genes. For each cell type, differentially expressed genes were filtered at a fold change of 1.5 and P-adjusted value of 0.05.

### Western Blotting

Aortic tissues or cultured cells were lysed with radioimmunoprecipitation assay (RIPA) buffer (Beyotime, P0013B) containing the protease inhibitor phenylmethanesulfonyl fluoride (Beyotime, ST506) and phosphatase inhibitors (Beyotime, P1081) to extract proteins. The collected proteins were subjected to electrophoresis and then electroblotted onto polyvinylidene fluoride (PVDF) membranes. Then, the membranes were blocked with 5% BSA in Tris-buffered saline with Tween 20 (TBST) at 37 °C for 1 h and incubated with primary antibodies at 4 °C overnight. The primary antibodies used for Western blotting were anti-p16, anti-p21, anti-p53, anti-γH2AX, anti-SM22α, anti-αSMA, anti-vimentin, anti-PDGFRβ, anti-ERK, anti-pERK anti-β-actin and anti-GAPDH at the corresponding dilutions. The membranes were washed with TBST and incubated with secondary antibodies (goat anti-mouse, sc-2005, Santa Cruz; goat anti-rabbit, sc-2004, Santa Cruz) at room temperature for 1 hour. Protein bands were finally visualized via enhanced chemiluminescence (Advance, No. RPN2235, GE Healthcare Life Sciences). Western blotting was replicated at least three independent times and quantified with ImageJ software. The intensity values were normalized to those of β-actin or GAPDH for total proteins. The following primary antibodies were employed (dilution): p16 (1:500; ab189034, Abcam), p21 (1:2000; 10355-1-AP, Proteintech), p21 (1:1000; ab188224, Abcam), p53 (1:5000; 10442-1-AP, Proteintech), γH2AX (1:500; sc-517348, Santa), SM22α (1:1000; 10493-1-AP, Proteintech), αSMA (1:3000; ab7817, Abcam), vimentin (1:2000; 10366-1-AP, Proteintech), pERK (1:1000; #9101, CST), ERK (1:1000; #9102, CST), PDGFRβ (1:1000; ab69506, Abcam), β-actin (1:1000; 20536-1-AP, Proteintech) and GAPDH (1:5000; 10494-1-AP, Proteintech).

### Enzyme-Linked Immunosorbent Assay (ELISA)

FGF9 concentrations in cell supernatants were determined by ELISAs. An FGF9 ELISA kit (SEA036Mu; Cloud-Clone Corp) was used according to the manufacturer's protocols. Optical density values were obtained at 450 nm using an ELISA plate reader (Spectra Max M5, Molecular Devices, California, United States).

### RNA Interference and Cell Transfection

Specific siRNA sequences against FGF9 and PDGFRβ were synthesized by Tsingke (Guangzhou, China; sequences of siRNAs are available in Supplementary Table 3). Primary VSMCs were seeded in 6-well plates and cultured for 24 hours. The cells were then starved with DMEM without FBS or penicillin/streptomycin. Then, 50 nmol/L siRNA and Lipofectamine 3000 (L3000015, Invitrogen) were added to two Eppendorf tubes with 250 µL of Opti-Minimum Essential Medium (Gibco BRL, Paisley, United Kingdom). Then, the solutions in the two tubes were mixed and incubated at room temperature for 20 minutes and added to the cells. After a 6-hour incubation, the medium was replaced with DMEM supplemented with FBS. The cells were finally subjected to RNA isolation, protein isolation or coculture experiments.

### Statistical Analysis

In our study, the elastin degradation grades are expressed as medians and quartiles, and the remaining quantitative values are presented as the mean±SD. A normal distribution test (Shapiro-Wilk test) was performed for continuous variables. After confirmation of the variance equality among different groups, Student's t test was used for the analysis of significant differences between two groups, whereas one-way ANOVA with a post Bonferroni's multiple comparisons test was used for the analysis between multiple groups. If variables were determined to be non-normally distributed, a nonparametric Kruskal-Wallis test with post Dunn's multiple comparisons test for multiple independent groups was applied. The aortic incidence between the groups was compared using Fisher's exact test. The log-rank (Mantel-Cox) test was used for survival analysis. The data were analyzed using SPSS, version 26.0 (SPSS, Inc., Chicago, IL) and GraphPad Prism 8 (GraphPad, USA). P < 0.05 was considered to be substantially significant.

## RESULTS

### SIPS occurs in human AAA tissues

**Figure 1.**
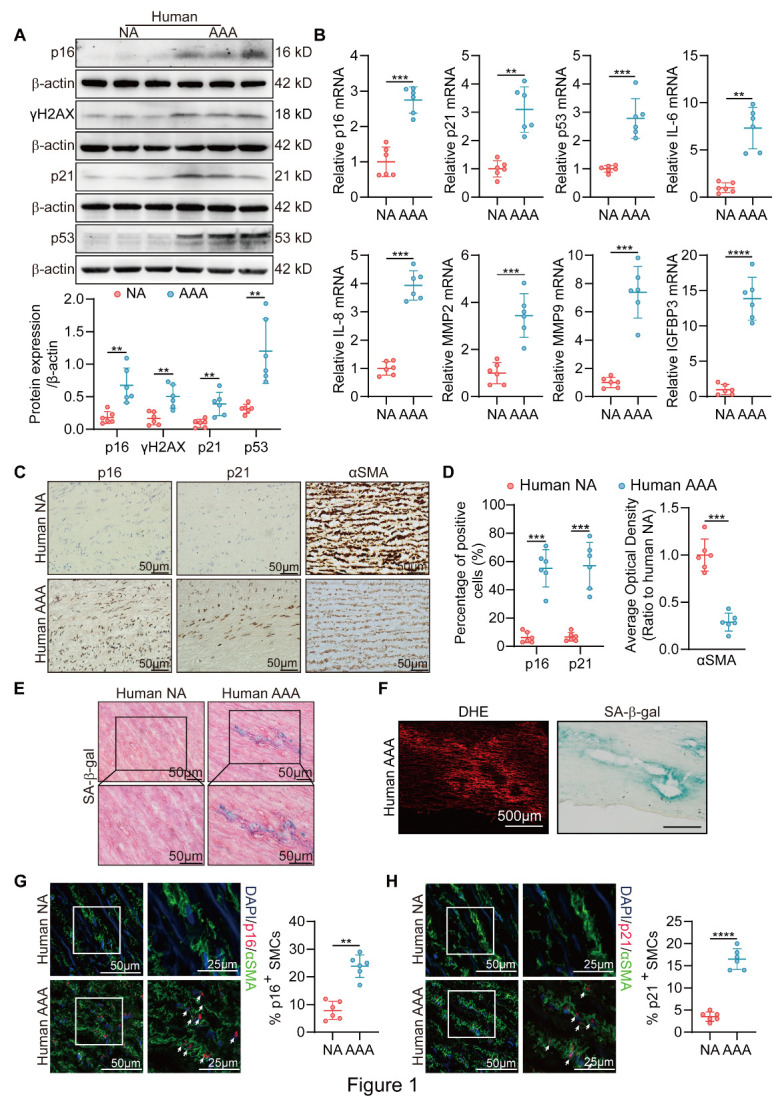
Markers of SIPS in human AAA. (A) Western blotting (WB) analysis of the protein levels of p16, γH2AX, p21, and p53 in human abdominal aortic aneurysms (AAA) and adjacent nonaneurysmal aortic (NA) sections (n = 6/group). (B) RT-PCR assessment of p16, p21, p53, IL-6, IL-8, MMP2, MMP9, and IGFBP3 in human AAA and NA samples (n = 6/group). RNA levels were normalized to β-actin. (C-D) Representative immunohistochemical staining and densitometric analysis for p16, p21 and αSMA in human AAA tissues and adjacent control aortas. The magnified photographs were taken at the location where the most severe aortic dilation occurred (scale bars = 50 μm; n = 6/group). (E) Representative images of the SA-β-gal assay in human AAA and NA sections (scale bars = 50 μm). (F) Representative image of DHE and SA-β-gal staining in the same AAA samples from young patients (scale bars = 500 μm). (G-H) Immunofluorescence staining analysis for αSMA (green) and p16 or p21 (red) signals in human AAA and NA aortas (scale bars = 50 and 25 μm; n = 6/group). Parametric paired t test for Fig. 1A-D and 1G-H. *P < 0.05, **P < 0.01, ***P < 0.001, ****P < 0.0001.

We first examined whether SIPS is related to the pathogenesis of human AAA. To distinguish SIPS from age-related senescence [36], we retrospectively collected human AAA sections and their control adjacent aortic sections from young patients aged less than or equal to 50 years old with risk factors and stress stimuli such as hyperlipidemia, hypertension and smoke. Western blotting results showed that the SIPS-associated markers p16, p21, p53 and the DNA damage response (DDR) marker γH2AX (gamma phosphorylated form of histone H2AX) were substantially higher in the human AAA tissues than in the corresponding adjacent tissues from the young patients (Fig. 1A). We also found that the mRNA levels of p16, p21, p53 and SASP markers, including IL-6, IL-8, MMP2, MMP9 and IGFBP3, were increased in human AAA tissues (Fig. 1B). In addition, immuno-histochemistry showed that the expression of p16 and p21 was significantly increased, while the expression of the VSMC marker α-SMA was decreased in human AAA (Fig. 1C-D). The SA-β-gal assay revealed that cells within the medial layer of aneurysmal tissues had higher SA-β-gal activity (Fig. 1E). To better determine the existence of SIPS, we newly performed DHE staining, an indicator of ROS, in the aneurysm samples and the adjacent tissues from young patients. We found that aneurysm sections showed higher ROS level (Supplementary Fig. 1), while the region displaying highest levels of oxidative stress overlapped with highest senescence burden (Fig. 1F). Immunofluorescence staining further showed that the human AAA tissues had a higher ratio of p16- and p21-positive VSMCs than the control tissues (Fig. 1G-H). We also detected senescence-associated markers p16, p21 and DNA damage response marker, γH2AX in the elderly AAA patients’ samples. We observed that the protein level of p16, p21 and γH2AX increased in human AAA tissues than in corresponding adjacent tissues in patients older than 65 years old (Supplementary Fig. 2A). Similarly, AAA tissues from elderly patients showed higher SA-β-gal activity than the adjacent tissues (Supplementary Fig. 2B). Moreover, we performed DHE staining in these aneurysm samples and the adjacent tissues. Our data showed higher levels of oxidative stress induced damage in aneurysm samples from elderly patients (Supplementary Fig. 2C). Meanwhile, the area showing higher oxidative stress displayed the highest SA-β-gal activity in AAA tissues obtained from elderly patients (Supplementary Fig. 2D). These results suggested that SIPS might also contribute to the AAA formation in elderly patients. In general, these results indicated that SIPS is involved in human AAA formation.

**Figure 2.**
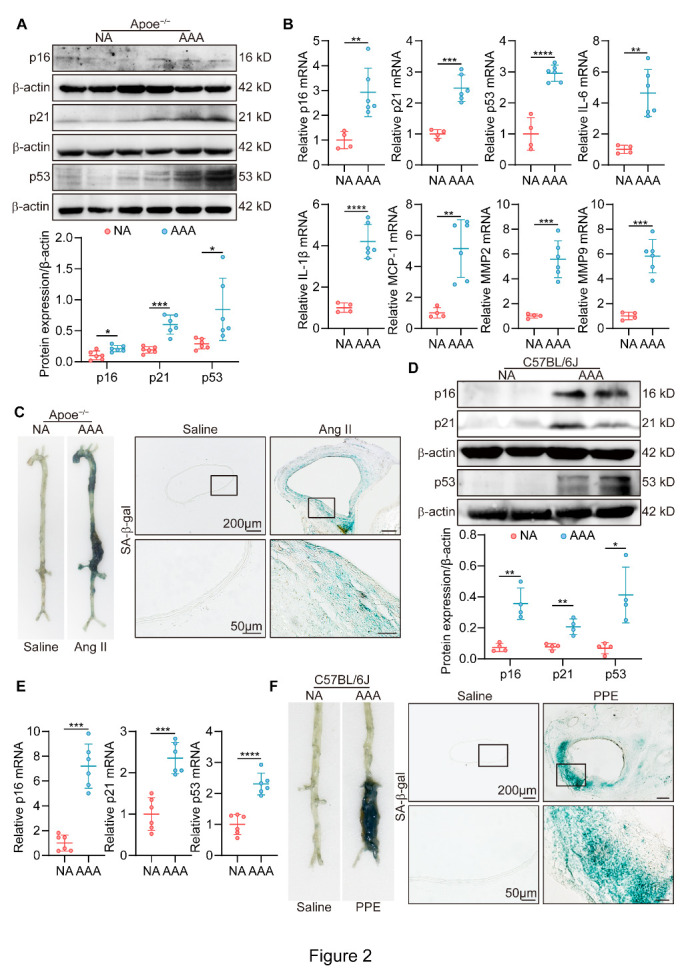
Experimental abdominal aortic aneurysms display SIPS. (A) WB analysis of p16, p21 and p53 in Ang II-infused ApoE^-/-^ mice (AAA) and saline-infused ApoE^-/-^ mice (NA) (n = 6/group). (B) Expression of SIPS markers and SASP factors measured by RT-PCR in Ang II-induced male ApoE^-/-^ mice (n = 6) and control male ApoE^-/-^ mice (n = 4). RNA levels were normalized to GAPDH. (C) Representative images of SA-β-gal assay in Ang II-treated model and control group (scale bars = 200 and 50 µm). (D) WB analysis of the protein levels of p16, p21 and p53 in the aortas from male C57BL/6J mice treated with elastase (AAA) or saline (NA) (n = 4/group). (E) RT-PCR analysis of cellular senescence markers (p16, p21 and p53) in elastase-induced AAA samples and saline-treated NA samples (n = 6/group). RNA levels were normalized to GAPDH. (F) Representative images of SA-β-gal assay in elastase-treated and saline-treated C57BL/6J mice (scale bars = 200 and 50 µm). Parametric unpaired t test for Fig. 2A-B and 2D-E. *P < 0.05, **P < 0.01, ***P < 0.001, ****P < 0.0001.

### Mouse AAA models exhibit SIPS

Next, we established Ang II-induced and elastase-induced mouse AAA models (Supplementary Fig. 3A-B) in nine- to twelve-week-old male mice to further confirm the role of SIPS in AAA formation with minimum age influence. Western blotting showed that p16, p21, and p53 were upregulated in the Ang II-induced AAA model mice compared with the control mice (Fig. 2A). In addition, the mRNA levels of p16, p21, p53 and the SASP, including IL-6, IL-1β, MCP-1, MMP2 and MMP9, were increased in the Ang II-induced AAA model mice (Fig. 2B). Moreover, we found that SA-β-gal-positive staining was mainly located in the media of aortas from Ang II-treated AAAs, suggesting an important role of SIPS in medial VSMCs (Fig. 2C). Concordant with the Ang II infusion model, exposure to elastase in male C57BL/6J mice also resulted in higher expression levels of SIPS markers and SASP factors and higher SA-β-gal activity (Fig. 2D-F and Supplementary Fig. 3C). We then analyzed the scRNA-seq data (GSE186865) and found that cellular senescence biomarkers, including Glb1, Cdkn1a and Trp53, were increased in Ang II-induced mouse AAA models (Supplementary Fig. 4A). Furthermore, the AUCell package showed that VSMCs had the highest senescence level, suggesting that VSMCs were the main cell type of SIPS in AAA models (Supplementary Fig. 4B-D). Taken together, these results demonstrated that SIPS of VSMCs might play an important role in AAA formation.

**Figure 3.**
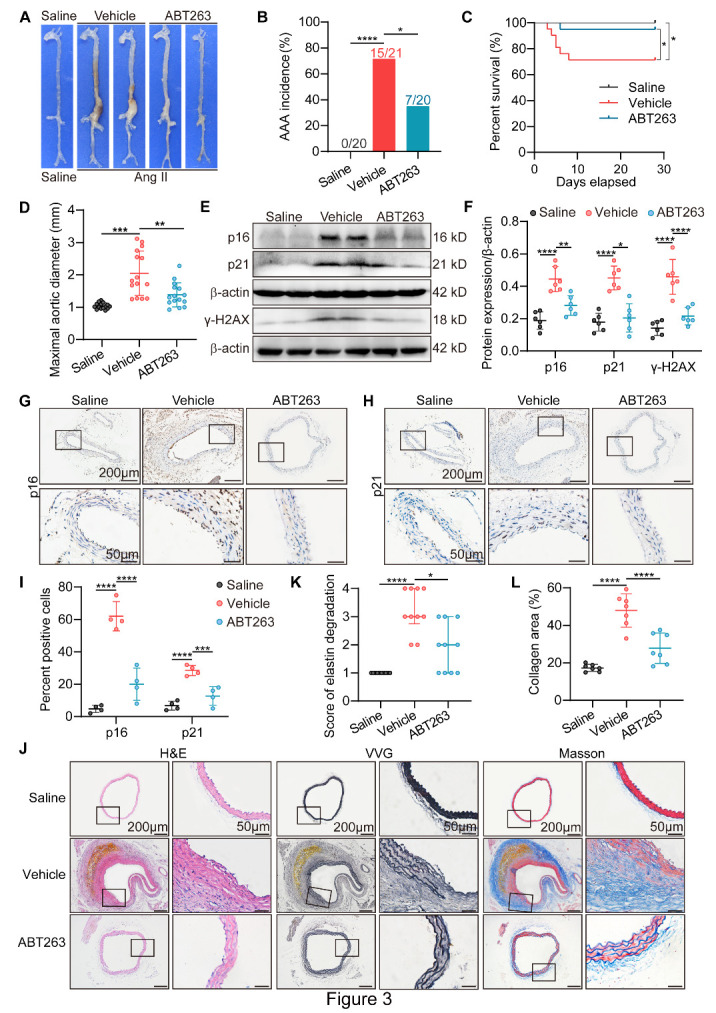
Removal of stress-induced premature senescent cells by ABT263 prevents Ang II-induced AAA formation and related pathological changes. All mice were infused with saline (Saline) or Ang II for 4 wk. Ang II-infused mice were randomly allocated to Vehicle group or ABT263 group and administered orally with vehicle or ABT263 (50 mg/kg) daily from 1 day post Ang II infusion for 14 consecutive days and every other day in the last two weeks. (A) Representative photographs showing macroscopic features of aneurysms induced by Ang II and ABT263 administration. (B) The AAA incidence in Saline group (n = 20), Vehicle group (n = 21) and ABT263 group (n = 20). (C) The survival curve in Saline group (n = 20), Vehicle group (n = 21) and ABT263 group (n = 20). (D) The maximal abdominal aortic diameter in the indicated groups (n = 15/group). (E-F) Western blotting and densitometric analysis of the protein level of p16, γH2AX and p21 in aorta homogenates (n = 6/group). (G-I) Representative densitometric analysis and staining of the p16 and p21 protein (scale bars = 200 and 50 µm; n = 4/group). (J) Representative hematoxylin and eosin (H&E), Verhoeff-Van Gieson (VVG), and Masson trichrome staining of the mouse aortas (scale bars = 200 and 50 µm). (K) Elastin degradation score in aortas (n = 10/group). Elastin degradation scores are shown for score 1, score 2, score 3, score 4, and expressed as medians and quartiles. The error bar represents the upper quartile and the lower quartile. (L) Percentage of collagen area in aortas (n = 7/group). Fisher's exact test for Fig. 3B, log-rank (Mantel-Cox) test for Fig. 3C, one-way ANOVA with a post Bonferroni's multiple comparisons test for Fig. 3D-I and 3L, and nonparametric Kruskal-Wallis test with post Dunn's multiple comparisons test for Fig. 3K. *P < 0.05, **P < 0.01, ***P < 0.001, ****P < 0.0001.

### ABT263 eliminates stress-induced premature senescent VSMCs and attenuates SASP

A previous study showed that ABT263 (navitoclax), targeting the antiapoptotic pathway BCL2 in senescent cells, could kill senescent mouse VSMCs [14]. ABT-263 is a potent and orally bioavailable Bad-like BH3 mimetic (Ki's of <1 nmol/L for Bcl-2, Bcl-xL, and Bcl-w), which disrupts Bcl-2/Bcl-xL interactions with pro-death proteins (e.g., Bim) and leads to the initiation of apoptosis [37]. In addition, ABT263 is being tested in clinical trials, and safety profiles have been demonstrated [38]. We therefore examined the ability of ABT263 (chemical structure shown in Supplementary Fig. 5A), the most widely studied senolytic [14], to remove stress-induced premature senescent VSMCs in vitro prior to assessing its effect on AAA. To further investigate whether ABT263 inhibits SIPS in vitro, we isolated primary mouse VSMCs (Supplementary Fig. 5B) and incubated mouse VSMCs with Ang II to induce SIPS in vitro. VSMCs treated with Ang II showed a significantly increased number of SA-β-gal-positive cells compared with the controls, whereas ABT263 selectively killed stress-induced premature senescent VSMCs in a dose-dependent manner (Supplementary Fig. 5C-D). Western blotting analysis and qPCR showed substantially increased protein levels of p16, p21, γH2AX and mRNA levels of p16, p21, p53 and SASP factors (including IL-6, IL-8, IL-1β, MCP-1, MMP2 and MMP9) in Ang II-induced premature senescent VSMCs, and these changes were reversed robustly after ABT263 treatment (Supplementary Fig. 5E-H). Furthermore, the SA-β-gal assay results showed that normal primary VSMCs exhibited cellular senescence upon coculture with VSMCs undergoing SIPS, while elimination of stress-induced premature senescent VSMCs through ABT263 alleviated the transmission of cellular senescence (Supplementary Fig. 5I-J). Collectively, these findings indicated that ABT263 substantially reduces stress-induced premature senescent VSMCs, SASP, and senescence transmission.

### ABT263 suppresses Ang II-induced AAA formation and related pathological changes in ApoE^-/-^ mice by inhibiting SIPS

We further explored whether intervention with SIPS by ABT263 affects AAA formation. On the first day post-Ang II infusion, mice were treated with either vehicle only or ABT263 for 14 consecutive days in the first two weeks and every other day in the last two weeks (Supplementary Fig. 6A). Our results showed that ABT263 had no influence on systolic blood pressure under Ang II infusion (Supplementary Fig. 6B). Ang II infusion for 4 weeks caused AAAs in 71.4% (15/21) of ApoE^-/-^ mice, whereas only 35.0% (7/20) of ApoE^-/-^ mice administered ABT263 orally developed AAAs after Ang II infusion (Fig. 3A-B). During the Ang II treatment period, vascular ultrasound imaging showed milder dilation in the abdominal aorta in the ABT263 group than in the vehicle group on the 14th day (Supplementary Fig. 6C). Moreover, ABT263 treatment strongly decreased the mortality caused by aortic rupture in the Ang II-treated mice (Fig. 3C). The maximal abdominal aortic diameter of the ABT263 administration group was substantially lower than that of the vehicle-treated mice (Fig. 3D). The protein expression of the SIPS markers p16, γH2AX, and p21 was substantially increased in the vehicle group after Ang II infusion and reversed by ABT263 treatment (Fig. 3E-F). Correspondingly, Ang II infusion induced the deposition of stress-induced premature senescent cells, as indicated by p16- and p21-positive cells in the media of the aortas, while ABT263 administration successfully eliminated stress-induced premature senescent cells (Fig. 3G-I). A significant difference in SA-β-gal-positive cells was also observed between the vehicle and ABT263 groups (Supplementary Fig. 6D). Moreover, Ang II infusion increased the mRNA expression of SASP factors, while ABT263 treatment reversed this effect (Supplementary Fig. 6E). In addition, the elastin degradation score and collagen content were substantially lower in the ABT263 group than in the vehicle group (Fig. 3J-L). Overall, we demonstrated that inhibition of SIPS by ABT263 ameliorates Ang II-induced AAA formation and related pathological changes, possibly by eliminating stress-induced premature senescent cells and reducing SASP factor expression.

As ABT263 targets the Bcl-2 family proteins, we also detected the expression levels of the related genes in AAA tissues. By analyzing the transcriptome data (GSE 57691[39]), we found that the expression of Bcl-2 family was not significantly different between human AAA tissues and normal tissues (Supplementary Fig. 7A-C). Then, we detected Bcl2 protein expression level in human AAA tissues by immunohistochemical staining and found that Bcl2 protein expression level was not significantly elevated in AAA tissues (Supplementary Fig. 7D-E), which was in accord with the transcriptome data results.

### Elimination of stress-induced premature senescent cells by ABT263 restrains elastase-induced AAA formation

**Figure 4.**
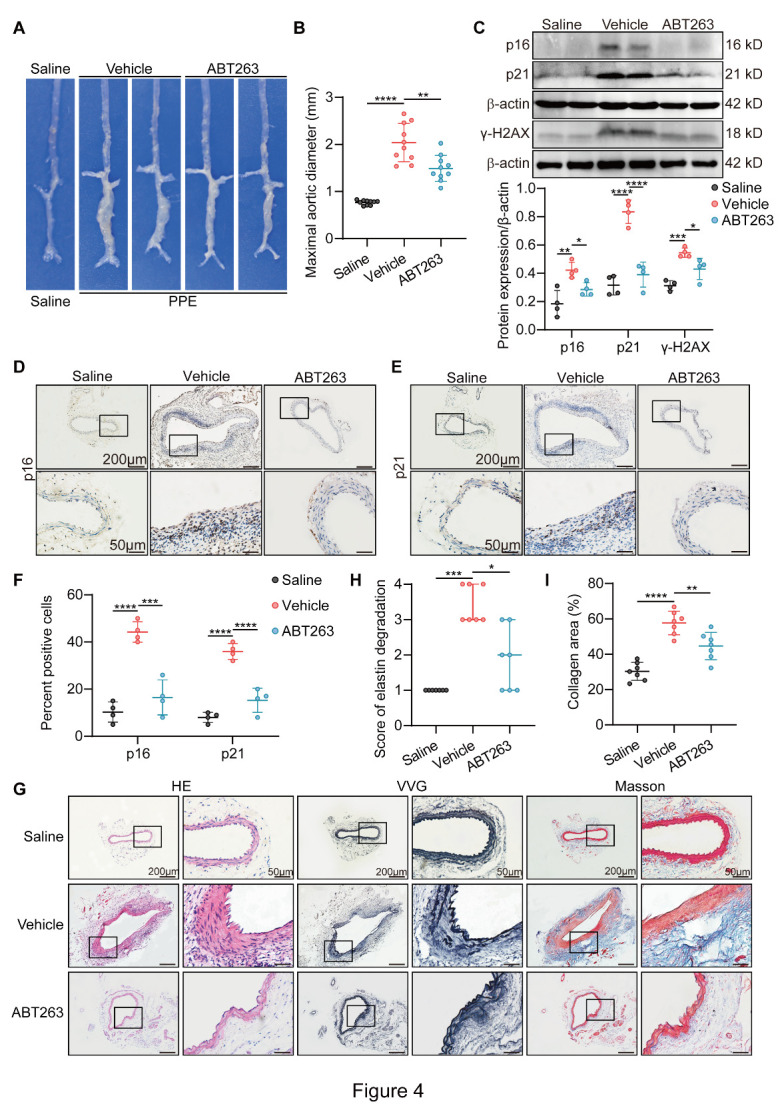
Clearance of stress-induced premature senescent cells restrains elastase-induced AAA formation and related vascular pathological changes. All mice were treated with saline (Saline) or elastase for 2 wk. Elastase-treated mice were randomly divided into Vehicle group and ABT263 group and treated orally with vehicle or ABT263 (50 mg/kg) daily for two weeks. (A) Representative photographs showing macroscopic features of aneurysms induced by elastase in wild-type (WT) mice for 2 wk. (B) The maximal abdominal aortic diameter of infrarenal aortas in indicated groups (n = 10/group). (C) Western blotting and densitometric analysis of the protein levels of p16, γH2AX and p21 in aorta homogenates (n = 4/group). (D-F) Representative immunohistochemical staining and analysis in aorta sections showing the expression of p16 and p21 in indicated groups (scale bars = 200 and 50 µm; n = 4/group). (G) Representative hematoxylin and eosin (H&E), Verhoeff-Van Gieson (VVG), and Masson trichrome staining of the mouse aortas (scale bars = 200 and 50 µm). (H) Elastin degradation score in aortas from indicated groups (n = 7/group). (I) Analysis of collagen area in aortas from different groups (n = 7/group). One-way ANOVA with a post Bonferroni's multiple comparisons test for Fig. 4B-F and 4I, and nonparametric Kruskal-Wallis test with post Dunn's multiple comparisons test for Fig. 4H. *P < 0.05, **P < 0.01, ***P < 0.001, ****P < 0.0001.

To further investigate whether the effect of elimination of stress-induced premature senescent cells by ABT263 on AAA formation is independent of Ang II, we generated elastase-induced AAA in C57BL/6J mice. In the established AAA, arteries presented severe dilation in the aortic lumen, whereas ABT263 unambiguously alleviated the dilation (Fig. 4A-B). In addition, the protein expression of p16, p21, and γH2AX and p16- and p21-positive stress-induced premature senescent cells in aortic media showed increasing trends after elastase treatment (Fig. 4C-F). Interestingly, the increased expression of the SIPS markers above was attenuated by ABT263 (Fig. 4C-F). Moreover, elastase exacerbated elastin degradation and collagen deposition in the aortic wall (Fig. 4G-I). Consistent with the results in Ang II-infused AAA, elastin disruption and collagen deposition were also rescued by ABT263 treatment (Fig. 4G-I). Taken together, these results corroborated the protective role of SIPS inhibition in elastase-induced AAA formation.

### Suppression of SIPS by BPTES protects against AAA development in ApoE^-/-^ mice

**Figure 5.**
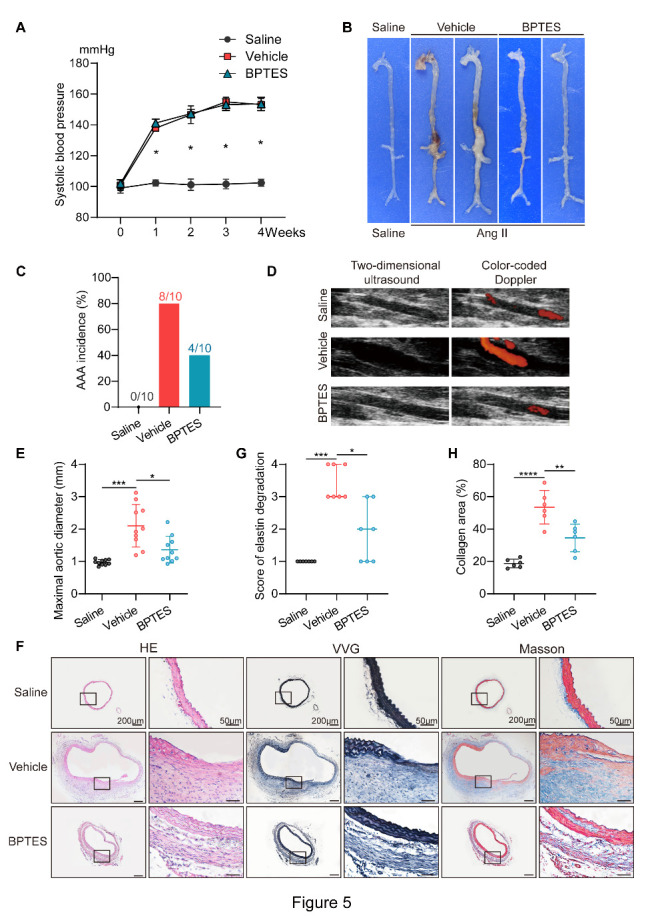
Inhibition of SIPS by BPTES suppresses Ang II-induced AAA formation. All male ApoE^-/-^ mice were infused with saline (Saline) or Ang II for 4 wk. AngII-infused mice were randomly assigned to Vehicle group or BPTES group and intraperitoneally injected with vehicle (200 µl of 10% DMSO in corn oil) or BPTES (0.25 mg/20 g/200 µl) three times a week for 1 month. (A) Systolic blood pressure at baseline and the indicated times after Ang II infusion (n = 7/group). *P < 0.05 Vehicle vs. Saline at each time point. (B) Representative photographs showing macroscopic features of aneurysms induced by Ang II. (C) The AAA incidence in Saline group, Vehicle group and BPTES group (n = 10/group). (D) Two-dimensional color-coded ultrasound imaging of aortic aneurysms after 14 d of Ang II or saline treatment. (E) The maximal abdominal aortic diameter in Saline group, Vehicle group and BPTES group (n = 10/group). (F) Representative H&E, VVG, and Masson trichrome staining of the mouse aortas from the 3 groups. Photographs showed the location where the most severe elastin degradation occurred (scale bars = 200 and 50 µm). (G) Elastin degradation score in aortas from indicated groups (n = 7/group). (H) Collagen area in aortas from the 3 groups (n = 6/group). One-way ANOVA with a post Bonferroni's multiple comparisons test for Fig. 5A, 5E and 5H, and nonparametric Kruskal-Wallis test with post Dunn's multiple comparisons test for Fig. 5G. *P < 0.05, **P < 0.01, ***P < 0.001, ****P < 0.0001.

BPTES [bis-2-(5-phenylacetamido-1,2,4-thiadiazol-2-yl)ethyl sulfide] is a potent inhibitor of glutaminase, inhibiting the allosteric activation caused by phosphate binding and promoting the formation of an inactive complex [40]. BPTES was reported to clear senescent cells through the glutaminolysis pathway, and BPTES treatment ameliorated senescence-associated vascular diseases such as atherosclerosis[41]. We explored whether elimination of stress-induced premature senescent cells by BPTES, independent of the BCL2 pathway, could still inhibit the occurrence and development of aneurysms. In our study, no significant difference in the systolic blood pressure of mice was observed between the vehicle group and the BPTES group (Fig. 5A). BPTES administration decreased the incidence of AAA (40.0%) compared with vehicle treatment (80.0%) (Fig. 5B-C). Additionally, the mice injected with BPTES showed attenuation in maximal abdominal aortic diameter measured by two-dimensional color-coded ultrasound imaging and macroscopic features (Fig. 5D-E). Furthermore, Van Gieson staining, and Masson’s trichrome staining confirmed that the disruption of elastic lamina structures and deposition of collagen in mouse AAA were prevented by BPTES (Fig. 5F-H).

### ABT263 attenuates AAA development by preventing VSMC phenotypic switching

**Figure 6.**
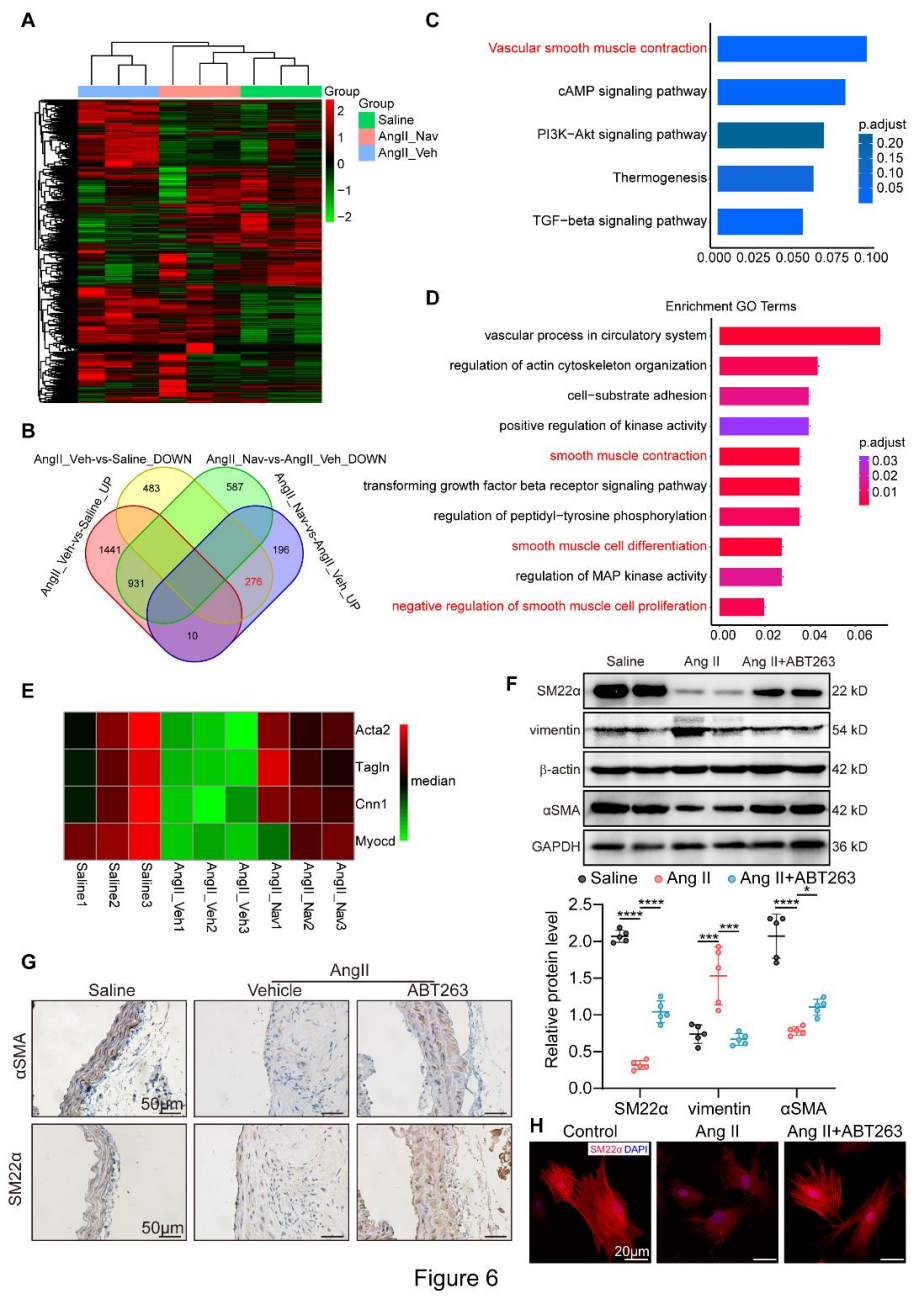
ABT263 attenuates AAA formation by preventing VSMC phenotypic switching. Apoe^-/-^ mice were either subjected to Ang II infusion or saline infusion (Saline). Ang II-infused Apoe^-/-^ mice were randomly assigned to Ang II_Veh or Ang II_Nav group and provided with vehicle or ABT263 (n = 3/group). (A) Hierarchical clustering heatmap illustrating the differentially expressed mRNAs in Saline, Ang II_Veh and Ang II_Nav groups. The map is color coded with red corresponding to upregulation and green to downregulation. (B) Venn diagram showing 276 differentially expressed genes that decreased in Ang II-perfused ApoE^-/-^ mice but increased following ABT263 treatment. (C) KEGG analysis using the selected 276 genes. (D) Gene Ontology analysis of the selected genes. (E) Heatmap about the SMC phenotypic switch markers including Acta2 (αSMA), Tagln (SM22α), Cnn1 (calponin1) and Myocd. (F) Western blotting analysis of the protein expression levels of VSMC phenotypic switching markers in abdominal aortas from saline-infused ApoE^-/-^ mice (Saline), Ang II-induced AAA mice administrated with Vehilce (Ang II) or ABT263 (Ang II+ABT263) (n = 5/group). (G) Representative immunohistochemical staining for αSMA and SM22α in suprarenal aortas from Saline group, Ang II group and Ang II+ABT263 group (scale bars = 50 μm). (H) Immunofluorescence staining for DAPI (blue) and SM22α (red) in VSMCs from indicated groups (scale bars = 20 µm). One-way ANOVA with a post Bonferroni's multiple comparisons test for Fig. 6F. *P < 0.05, ***P < 0.001, ****P < 0.0001.

We next conducted RNA sequencing to investigate the underlying role of SIPS in AAA. In total, 2572 mRNAs were differentially expressed in the Ang II_Veh group compared with the saline group, while 1449 mRNAs were differentially expressed in the Ang II_Nav group compared with the Ang II_Veh group (Fig. 6A). To identify the functional pathways modulated by ABT263, we identified 276 differentially expressed genes, which were downregulated after Ang II infusion but increased following ABT263 treatment (Fig. 6B). Kyoto Encyclopedia of Genes and Genomes (KEGG) analysis of the 276 selected genes showed that the top enriched functional pathway was associated with vascular smooth muscle contraction (Fig. 6C). Additionally, Gene Ontology analysis revealed that the 276 genes encompassed genes regulating smooth muscle contraction, smooth muscle cell differentiation and negative regulation of smooth muscle cell proliferation (Fig. 6D). In particular, myocardin and VSMC contractile genes, including Acta2, Tagln, and Cnn1, were downregulated in AAA samples and upregulated following ABT263 treatment (Fig. 6E). These results indicate the essential effect of SIPS on VSMC phenotypic switching.

In vivo, qPCR indicated reduced levels of the VSMC contractile markers αSMA, SM22α, Myh11 and calponin1 in Ang II-induced AAA, which were normalized by ABT263 (Supplementary Fig. 8A). Moreover, Western blotting revealed that ABT263 countered Ang II-induced undesirable changes in αSMA, SM22α and vimentin (Fig. 6F). Consistently, immunostaining assay indicated reduced levels of VSMC contractile markers αSMA and SM22α after Ang II treatment, the effects of which were normalized by ABT263 (Fig. 6G). Moreover, qPCR and immunohistochemistry staining revealed that ABT263 reversed elastase-induced VSMC phenotypic switching (Supplementary Fig. 8B-C). Immunofluorescence staining of VSMCs revealed decreased myofilament formation and SM22α expression in response to Ang II stimulation, whereas ABT263 administration reversed SM22α expression (Fig. 6H). The unbiased findings demonstrated that inhibition of SIPS by ABT263 administration prevents VSMC phenotypic switching in AAA.

### ABT263 prevents the phenotypic switching of VSMCs via SASP reduction

**Figure 7.**
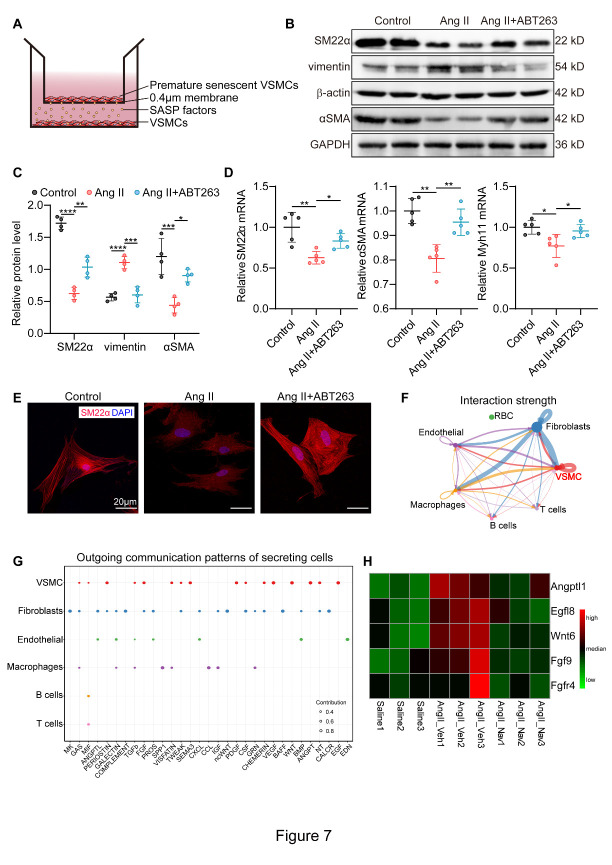
ABT263 blocks VSMC phenotypic switching by means of SASP reduction. (A) Diagrammatic representation of coculture experiments. (B-C) Western blotting of the protein expression levels of VSMC phenotypic switching markers in VSMCs cocultured with control VSMCs (Control), stress-induced premature senescent VSMCs (Ang II) and ABT263-treated premature senescent VSMCs (Ang II+ABT263) (n = 4/group). (D) mRNA expression levels of VSMC contractile markers in VSMCs from Control group, Ang II group and Ang II+ABT263 group (n = 5/group). RNA levels were normalized to GAPDH. (E) Immunofluorescence staining for DAPI (blue) and SM22α (red) in VSMCs from Control group, Ang II group and Ang II+ABT263 group (scale bars = 20 µm). (F) Circle plot showing the inferred intercellular communication network among different types of cells. The number of cells of each type is proportional to the circle size, and the line thickness represents the strength of interaction. (G) The secretory pattern of VSMCs obtained from scRNA-seq (GSE186865). (H) Heatmap analyzed from RNA-Seq results showing the expression of the secretory patterns of VSMCs (including Angptl1, Egfl8, Wnt6, Fgf9, Fgfr4). One-way ANOVA with a post Bonferroni's multiple comparisons test for Fig. 7B-D. *P < 0.05, **P < 0.01, ***P < 0.001, ****P < 0.0001.

Studies have revealed that stress-induced premature senescent cells exhibit a SASP program of inflammatory cytokines, chemokines, growth factors and matrix remodeling factors [42], which could alter the tissue environment and contribute to vascular diseases [36]. In addition, SASP has been implicated in modulating cellular phenotypic transformation [43]. We thus examined whether SASP reduction was responsible for the protective role of ABT263 in VSMC phenotypic switching in AAA. In our study, we cocultured normal VSMCs with nonsenescent VSMCs, stress-induced premature senescent VSMCs or ABT263-treated stress-induced premature senescent VSMCs to explore the role of SASP (Fig. 7A). We found that normal VSMCs cocultured with stress-induced senescent VSMCs demonstrated lower expression of the contractile genes SM22α and αSMA and higher levels of the synthetic gene vimentin than the control cells, and these changes could be substantially attenuated by decreasing the SASP through clearance of stress-induced premature senescent VSMCs, as shown by Western blotting (Fig. 7B-C). qPCR results also showed decreased mRNA levels of contractile genes (αSMA, SM22α and Myh11) and increased mRNA levels of synthetic genes (epiregulin, thro, Pcna and vimentin) in normal VSMCs cocultured with stress-induced premature senescent VSMCs; these effects could be reversed by ABT263 treatment (Fig. 7D and Supplementary Fig. 9). In addition, immunofluorescence staining of VSMCs revealed decreased myofilament formation and SM22α expression after coculture with stress-induced senescent VSMCs, whereas ABT263 administration increased SM22α expression (Fig. 7E). Altogether, these results suggested that ABT263 suppressed VSMC switching from a procontractile to a prosynthetic phenotype by reducing SASP factors, sequentially inhibiting AAA development.

**Figure 8.**
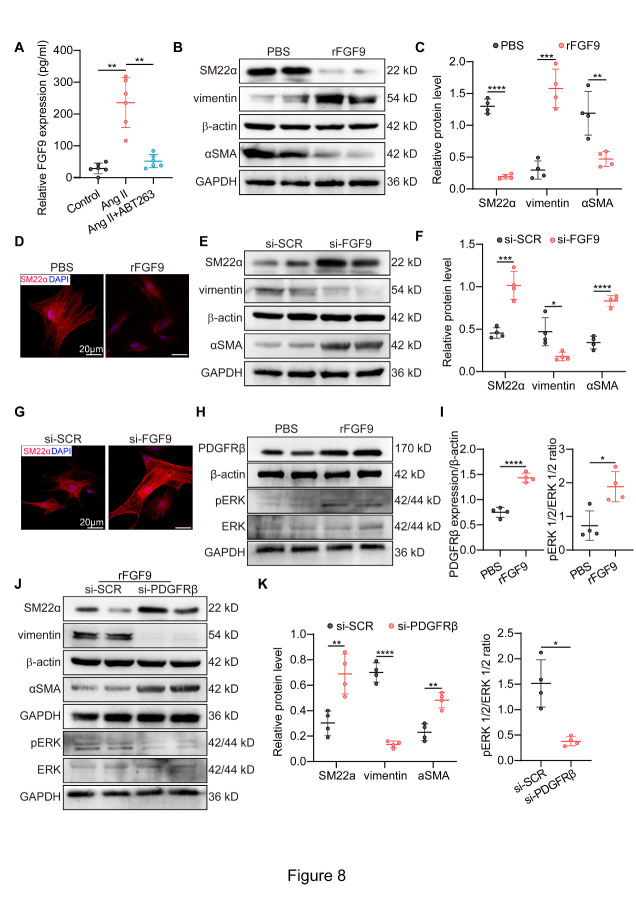
FGF9 works as the key SASP factor to mediate VSMC phenotypic switch. (A) FGF9 detection by enzyme-linked immunosorbent assay. FGF9 from VSMCs supernatants after incubation in control media, Ang II and Ang II+1.0 μM ABT263 (n = 6/group). (B-C) Western blotting of SM22α, αSMA, vimentin in VSMCs with or without exogenous rFGF9 treatment (n = 4/group). (D) Representative immunofluorescence staining in VSMCs treated by PBS or rFGF9 (scale bars = 20 µm). (E-F) Western blotting of SM22α, αSMA, and vimentin in VSMCs cocultured with Ang II-treated VSMCs with or without FGF9 knockdown (n = 4/group). (G) Immunofluorescence staining in VSMCs which were cocultured with Ang II-incubated VSMCs transfected with si-SCR or si-FGF9 (scale bars = 20 µm). (H-I) Western blotting of pERK, ERK and PDGFRβ in VSMCs with or without exogenous rFGF9 addition (n = 4/group). (J-K) Western blotting of SM22α, αSMA, vimentin, pERK, ERK in rFGF9-treated VSMCs with or without PDGFRβ knockdown (n = 4/group). One-way ANOVA with a post Bonferroni's multiple comparisons test for Fig. 8A, and parametric unpaired t test for Fig. 8B-C, 8E-F and 8H-K. *P < 0.05, **P < 0.01, ***P < 0.001, ****P < 0.0001.

### FGF9 functions as the key SASP factor to regulate VSMC phenotypic switching

We further investigated the key SASP factors leading to VSMC phenotypic switching. The ligand-receptor interaction-based strategy was used to uncover cell-cell communications in AAA based on single-cell RNA sequencing data (scRNA-seq, GSE186865 [35]), which exhibited strong interactions in VSMCs-VSMCs (Fig. 7F). Among the secretory patterns of VSMCs from scRNA-seq (Fig. 7G), several ligands, including Angptl1, Egfl8, Wnt6, Fgf9, and Fgfr4, were upregulated following Ang II infusion and decreased by ABT263 according to our RNA-seq results (Fig. 7H). As shown in previous studies, fibroblast growth factor 9 (FGF9) is a potent regulator of VSMC dedifferentiation, proliferation and migration among the five proteins mentioned above [44, 45]. In addition, FGF9 is critical in VSMC phenotypic modulation [45]. Meanwhile, previous studies have reported the close association between FGF receptors and VSMC phenotypic switch. It was uncovered that fibroblast growth factor receptor-1 (FGFR1) signaling is one of several signaling pathways involved in this VSMC phenotypic switching [46]. Additionally, FGF receptor substrate 2 (FRS2) down-regulation could ablate the facilitation effect of FGF signaling pathway on VSMC phenotypic switching [47]. These results indicated that FGF receptors play an important role in the VSMC phenotypic switch. Then, we focused on FGF9 to elucidate how stress-induced premature senescent VSMCs facilitate the phenotypic switch of surrounding normal VSMCs.

**Figure 9.**
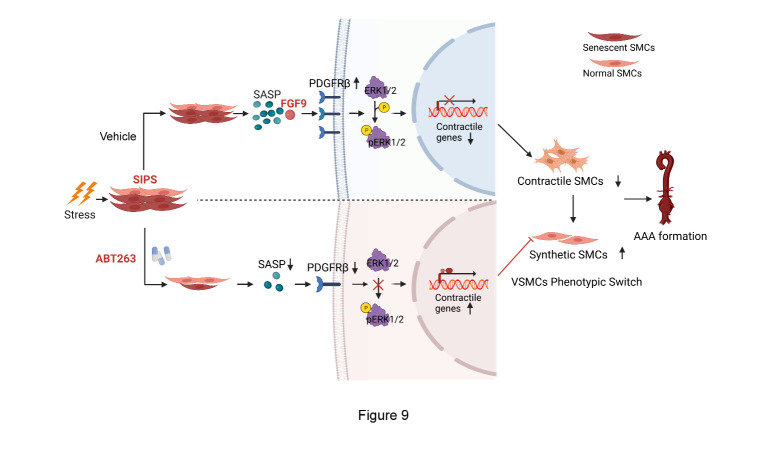
Schematic illustrating the mechanism of SIPS in AAA. Schematic illustrating that stress-induced premature senescent VSMCs result in the phenotypic switch of surrounding VSMCs through activating FGF9/PDGFRβ/ERK1/2 signaling, eventually promoting AAA formation, while eliminating stress-induced premature senescent cells by senolytics reverses the effect.

In our study, a notable increase in FGF9 was observed in the supernatant of VSMCs undergoing SIPS, which was strongly attenuated by ABT263 (Fig. 8A). Moreover, qPCR, Western blotting and immuno-fluorescence staining showed that the addition of recombinant FGF9 (rFGF9) protein could facilitate VSMC phenotypic switching (Fig. 8B-D and Supplementary Fig. 10A-B). We further constructed three different FGF9-targeting siRNAs to reduce FGF9 expression in vitro, of which FGF9 siRNA3 (>70% silencing effect) was the most effective with respect to knockdown efficiency (Supplementary Fig. 10C). FGF9 knockdown in senescent VSMCs rescued the effect of promoting phenotypic switch of the neighboring VSMCs (Fig. 8E-G and Supplementary Fig. 10D). These findings indicated that FGF9, secreted by stress-induced premature senescent VSMCs, is a pivotal SASP factor regulating the phenotypic switching of surrounding VSMCs. In addition, PDGFRβ/ERK1/2 signaling is significant for the regulation of VSMC behavior[48], and we explored whether PDGFRβ/ERK1/2 signaling was involved in the adverse effect of FGF9. Western blotting results showed increased expression of PDGFRβ and phosphorylation of ERK1/2 in VSMCs incubated with rFGF9 (Fig. 8H-I), while PDGFRβ knockdown reversed FGF9-induced ERK1/2 phosphorylation and VSMC phenotypic switching (Fig. 8J-K and Supplementary Fig. 10E-F). Collectively, these findings indicated that FGF9 is the key SASP factor for VSMC phenotypic switching through the PDGFRβ/ERK1/2 pathway (Fig. 9).

## DISCUSSION

In this study, we revealed that eliminating stress-induced premature senescent VSMCs could prevent the switch of normal VSMCs to an adverse phenotype, thus significantly delaying AAA progression. Mechanistically, stress-induced premature senescent VSMCs secreted FGF9, increasing PDGFRβ in surrounding normal VSMCs and eventually resulting in the phenotypic switch of normal VSMCs. Altogether, our findings demonstrated that the elimination of stress-induced premature senescent cells might be a promising preventive therapy for AAA.

One of the most important findings of our study is that SIPS plays an essential role during the pathogenic process of AAA, having great significance in AAA treatment. In our study, we found that stress-induced premature senescent cells substantially accumulated in the middle layer of AAA tissue in young human samples compared to adjacent control samples. We also observed that a portion of vascular cells showed signs of premature senescence in the aortas of young mice after Ang II or elastase application. These results indicated that SIPS in vascular cells was involved in the development and pathogenesis of AAA. Functional studies using two AAA mouse models further revealed that removing stress-induced premature senescent cells was sufficient to attenuate AAA formation, as shown by lower aortic diameter and mortality. Previous studies have demonstrated that aging-related senescence in vascular cells is responsible for AAA development in aging mice [33], which, combined with our results, could support the important role of cellular senescence in AAA formation. Notably, in contrast to age-related senescence, SIPS was closely associated with risk factors [13] for AAA, such as hypertension [10], hyperlipidemia [12] and cigarettes [11]. The above results indicated that SIPS might mediate the effect of risk factors on initiating the pathogenic process of AAA. Moreover, we confirmed the effectiveness and safety of ABT263 and BPTES in preventing or treating AAA by eliminating stress-induced premature senescent cells. In particular, both types of agents with different mechanisms, ABT263 and BPTES, were found to be useful in AAA treatment, demonstrating the stability of senolytics in alleviating AAA formation and progression. The current study thus suggests that inhibition of SIPS might be an attractive therapeutic approach to prevent or treat AAA.

Another interesting finding of our study was that SIPS escalated vascular wall vulnerability to dilatation and promoted AAA development, probably through the phenotypic switch of VSMCs. VSMCs are susceptible to switching from a contractile phenotype to a synthetic and inflammatory phenotype upon various stimuli [49]. Compelling evidence has indicated that VSMC phenotype switching is closely associated with the early onset of various vascular diseases [49], including AAA [50]. In this study, we found that SIPS could lead to the transformation of VSMCs from a procontractile to a prosynthetic phenotype, manifested by decreased expression of contractile markers and enhanced expression of synthetic markers, thus increasing the probability of aneurysm formation and rupture. Consistent with our study, recent research suggested that senescence might be responsible for the phenotypic change of nonsenescent cells through the SASP approach [43]. The link between SIPS and VSMC phenotypic switching further confirmed the role of SIPS in initiating the pathogenesis of AAA. More importantly, our findings are meaningful in the development of novel treatments for VSMC phenotypic switching. Despite the important role of VSMC phenotypic switching in AAA formation, there are still no proven therapies to inhibit or reverse VSMC phenotypic switching [51]. Our data showed that inhibition of SIPS by ABT263 could effectively repress VSMC phenotypic transition, thereby inhibiting chronic inflammation and elastin degradation in the aorta in vivo. These results demonstrated that senolytic therapy might be a promising therapeutic strategy to maintain VSMC homeostasis during vascular injury, providing a potent approach to treat a variety of vascular diseases in addition to AAA.

We further investigated the contribution of SIPS to VSMC phenotypic switching in terms of molecular mechanisms. Combined with the analysis of single-cell RNA sequencing and RNA sequencing, this study demonstrated that stress-induced premature senescent VSMCs secreted FGF9 to augment the expression of PDGFRβ and subsequent ERK1/2 phosphorylation, facilitating the phenotypic switching of cocultured VSMCs. Previous studies indicated that FGF9 plays a critical role in abnormal VSMC proliferation and migration [44], which is closely linked to the phenotypic switch of VSMCs. In our study, stress-induced premature senescent VSMCs secreted FGF9 to promote phenotypic switching of cocultured VSMCs, while the phenotypic switch of VSMCs was suppressed by the reduction in FGF9. Furthermore, our study found that FGF9 promotes the phenotypic transformation of VSMCs by upregulating PDGFRβ. FGF9 serves as a critical upstream regulator of PDGFRβ [45], and PDGFRβ exerts a significant effect on VSMC dedifferentiation and AAA development [52]. Moreover, this study demonstrated that increased PDGFRβ could lead to phosphorylation of ERK1/2. The increased phosphorylation levels of ERK1/2 mediate phenotypic switching of VSMCs [50]. Altogether, our data suggested that stress-induced premature senescent VSMC-derived FGF9 regulated PDGFRβ to modulate ERK1/2 phosphorylation, thus contributing to shifting the phenotype of VSMCs.

There are still some potential limitations in the current study. First, we used two types of agents to illustrate the effectiveness and safety of senolytic therapy in treating AAA. However, there are still other types of senolytic drugs that have been reported to inhibit SIPS [53]. Which type of senolytic drug is the most suitable agent for treating AAA should be explored in the future. In addition, our study revealed that stress-induced premature senescent cells secreted FGF9 to promote the phenotypic switch of VSMCs and AAA formation. Although we confirmed the important function of FGF9 by using siRNA in vitro, conditional gene knockout in stress-induced premature senescent cells should be performed to further determine the role of FGF9 during AAA development and progression.

In summary, our data showed that SIPS led to the accumulation of stress-induced premature senescent cells in the abdominal aorta, which promoted VSMC phenotypic switching by activating FGF9/PDGFRβ/ ERK1/2 signaling and eventually induced AAA formation. These findings suggested that targeting SIPS might be a powerful approach for treating AAA.

## Supplementary Materials

The Supplementary data can be found online at: www.aginganddisease.org/EN/10.14336/AD.2023.0215.



## Data Availability

The data that support the findings of this study are available from the corresponding author upon reasonable request.
