# Supplementary material for: Clearance of Stress-Induced Premature Senescent Cells Alleviates the Formation of Abdominal Aortic Aneurysms
Source: Aging Dis. 2023 Oct 1;14(5):1778–98. doi: 10.14336/AD.2023.0215 (PMC10529745; doi:10.14336/AD.2023.0215)
Supplement: Supplementary file 1 [file AD-14-5-1778-s.pdf]

SUPPLEMENTARY DATA

Clearance of Stress-Induced Premature Senescent Cells Alleviates the Formation of Abdominal Aortic Aneurysms

Jingfang Xie[#], Zhenquan Tang[#], Qiqi Chen, Xiaoqian Jia, Chuling Li, Ming Jin, Guoquan Wei, Hao Zheng, Xinzhong Li, Yanmei Chen, Wangjun Liao, Yulin Liao, Jianping Bin[*], Senlin Huang[*]

© 2022. Xie J et al. Published online at http://www.aginganddisease.org/EN/10.14336/AD.2023.0215

SUPPLEMENTARY DATA

Supplementary Table 1. Patient clinical information (n = 9).

Patient	Gender	Age	Smoke	Diameter (mm)	HL	HTN	CAD	Medication before surgery
ID1	male	47	no	60	yes	yes	no	bisoprolol, nifedipine, ambroxol, piperacillin/tazobactam, esomeprazole, recombinant human brain natriuretic peptide, atorvastatin
ID2	male	46	yes	57	no	yes	no	bisoprolol, urapidil, heamocoagulase agkistrodon, cefuroxime
ID3	male	47	no	57	no	yes	no	fentanyl, urapidil
ID4	male	40	yes	55	no	yes	no	fentanyl, urapidil, arotinolol, nifedipine, cefoperazone/sulbactam
ID5	male	50	yes	56	yes	yes	no	heamocoagulase agkistrodon, esomeprazole, piperacillin-tazobactam
ID6	male	50	yes	65	yes	yes	yes	pantoprazole, ambroxol
ID7	male	67	yes	47	no	yes	no	cefoperazone/sulbactam, ambroxol, pantoprazole
ID8	male	65	yes	52	no	yes	yes	cefoperazone/sulbactam, ambroxol, esomeprazole, atorvastatin, nifedipine
ID9	male	67	no	50	no	yes	no	piperacillin/tazobactam, alprostadil, ambroxol, recombinant human brain natriuretic peptide, pantoprazole

HL = Hyperlipidemia
HTN = Hypertension
CAD = Coronary artery disease

Supplementary Table 2. Primer sequences for quantitative real-time PCR (from 5’ to 3’).

p16 (human)	Forward	CCCAACGCACCGAATAGTTA
	Reverse	ACCAGCGTGTCCAGGAAG
p21 (human)	Forward	AGCGACCTTCCTCATCC
	Reverse	CCTCTACTGCCACCATCT
p53 (human)	Forward	GACTGACATTCTCCACTTCT
	Reverse	CTTCTGACGCACACCTATT
IL-6 (human)	Forward	GTGAGGAACAAGCCAGAG
	Reverse	TGACCAGAAGAAGGAATGC
IL-8 (human)	Forward	CGGAAGGAACCATCTCAC
	Reverse	CCACTCTCAATCACTCTCAG
MMP2 (human)	Forward	AAGCACAGCAGGTCTCA
	Reverse	AGCCAAGCGGTCTAAGT
MMP9 (human)	Forward	CGACGATGACGAGTTGT
	Reverse	AGGCGGAGTAGGATTGG
IGFBP3 (human)	Forward	CTTAGGCGGCTGTGTTG
	Reverse	TGAATGTGGAGGCTGACT
β-actin (human)	Forward	CCTTCCTGGGCATGGAGTC
	Reverse	TGATCTTCATTGTGCTGGGTG
p16 (mouse)	Forward	CCGATTCAGGTGATGATGAT
	Reverse	CGCACGATGTCTTGATGT
p21 (mouse)	Forward	TTCCTTGCCACTTCTTACC
	Reverse	ACTGCTTCACTGTCATCC
p53 (mouse)	Forward	ACAAGAAGTCACAGCACAT
	Reverse	ATAGGTCGGCGGTTCAT
IL-6 (mouse)	Forward	CCGCTATGAAGTTCCTCTC
	Reverse	GGTATCCTCTGTGAAGTCTC
IL-8 (mouse)	Forward	GAGACCTGAGAACAAGAGAA
	Reverse	ATCCATACACCAGACTAACG
MMP2 (mouse)	Forward	CTACACCTACACCAAGAACT
	Reverse	CCTCATACACAGCGTCAA
MMP9 (mouse)	Forward	GCGTGTCTGGAGATTCGACTTG
	Reverse	ACTGCAGGAGGTCGTAGGTCAC

© 2022. Xie J et al. Published online at http://www.aginganddisease.org/EN/10.14336/AD.2023.0215

SUPPLEMENTARY DATA

IL-1β (mouse)	Forward	CTTCAGGCAGGCAGTATC
	Reverse	CAGCAGGTTATCATCATCATC
MCP-1 (mouse)	Forward	CAATGAGTAGGCTGGAGAG
	Reverse	GAAGTGCTTGAGGTGGTT
IGFBP3 (mouse)	Forward	GGTCCTTATTGTGCCATCT
	Reverse	GGTCCTTATCCTTGTCCTTAT
SM22α (mouse)	Forward	GTGTGATTCTGAGCAAATTGGTG
	Reverse	ACTGCTGCCATATCCTTACCTT
αSMA (mouse)	Forward	GTCCCAGACATCAGGGAGTAA
	Reverse	TCGGATACTTCAGCGTCAGGA
calponin1 (mouse)	Forward	TCTGCACATTTTAACCGAGGTC
	Reverse	GCCAGCTTGTTCTTTACTTCAGC
Myh11 (mouse)	Forward	AAGCTGCGGCTAGAGGTCA
	Reverse	CCCTCCCTTTGATGGCTGAG
vimentin (mouse)	Forward	CGGCTGCGAGAGAAATTGC
	Reverse	CCACTTTCCGTTCAAGGTCAAG
thrombospondin (mouse)	Forward	GGGGAGATAACGGTGTGTTTG
	Reverse	CGGGGATCAGGTTGGCATT
epiregulin (mouse)	Forward	CTGCCTCTTGGGTCTTGACG
	Reverse	GCGGTACAGTTATCCTCGGATTC
Pcna (mouse)	Forward	TTTGAGGCACGCCTGATCC
	Reverse	GGAGACGTGAGACGAGTCCAT
GAPDH (mouse)	Forward	GGTATCCTCTGTGAAGTCTC
	Reverse	TTGCTGTTGAAGTCGCAGGAG

Supplementary Table 3. FGF9 and PDGFRβ siRNA sequences.

siRNA 1 FGF9, sense	5’-GGAGAUACUAUGUUGCAUUTT-3’
siRNA 2 FGF9, sense	5’-GGAGCUGUAUGGAUCAGAATT-3’
siRNA 3 FGF9, sense	5’-GUCCGGUGUUGCUAAGUGATT-3’
siRNA 1 PDGFRβ, sense	5’-GAUGUCACUGAGACGACAA-3’
siRNA 2 PDGFRβ, sense	5’-CCGAUACUUACUACGUCUA-3’
siRNA 3 PDGFRβ, sense	5’-CAGAGUUUGUUCUCAACAU-3’

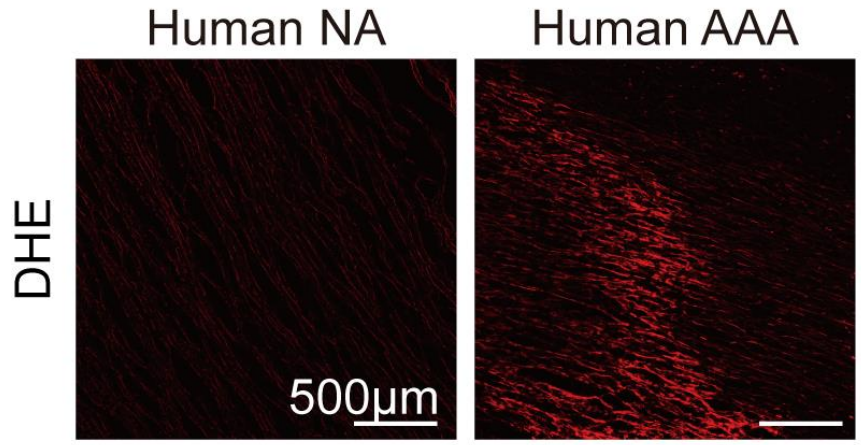

Supplementary Figure 1. Detection of ROS in human AAA tissues from young patients. Representative image of DHE staining in human AAA and NA tissues obtained from young patients (scale bars = 500 µm).

© 2022. Xie J et al. Published online at http://www.aginganddisease.org/EN/10.14336/AD.2023.0215

SUPPLEMENTARY DATA

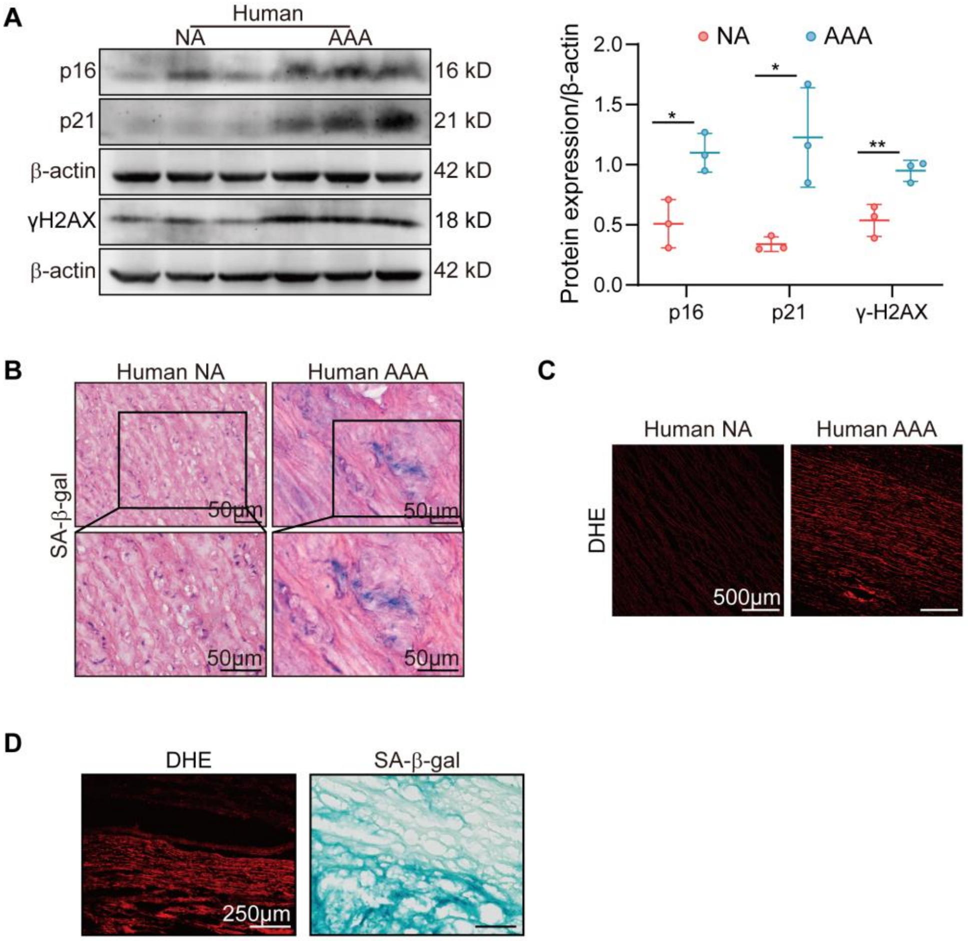

Supplementary Figure 2. Detection of SIPS in human AAA tissues obtained from elderly patients. (A) Western blotting analysis of the protein levels of p16, p21 and γH2AX in human abdominal aortic aneurysms (AAA) and adjacent nonaneurysmal aortic (NA) tissues obtained from elderly patients (n = 3/group). **(B)** Representative images of the SA-β-gal assay in human AAA and NA tissues obtained from elderly patients (scale bars = 50 μm). **(C)** Representative image of DHE staining in human AAA and NA tissues (scale bars = 500 μm). **(D)** Representative image of DHE and SA-β-gal staining from the same AAA sample (scale bars = 250 μm). Parametric paired t test for Supplementary Fig. 2A. $^{*}P < 0.05$, $^{**}P < 0.01$ vs. Human NA group.

© 2022. Xie J et al. Published online at http://www.aginganddisease.org/EN/10.14336/AD.2023.0215

SUPPLEMENTARY DATA

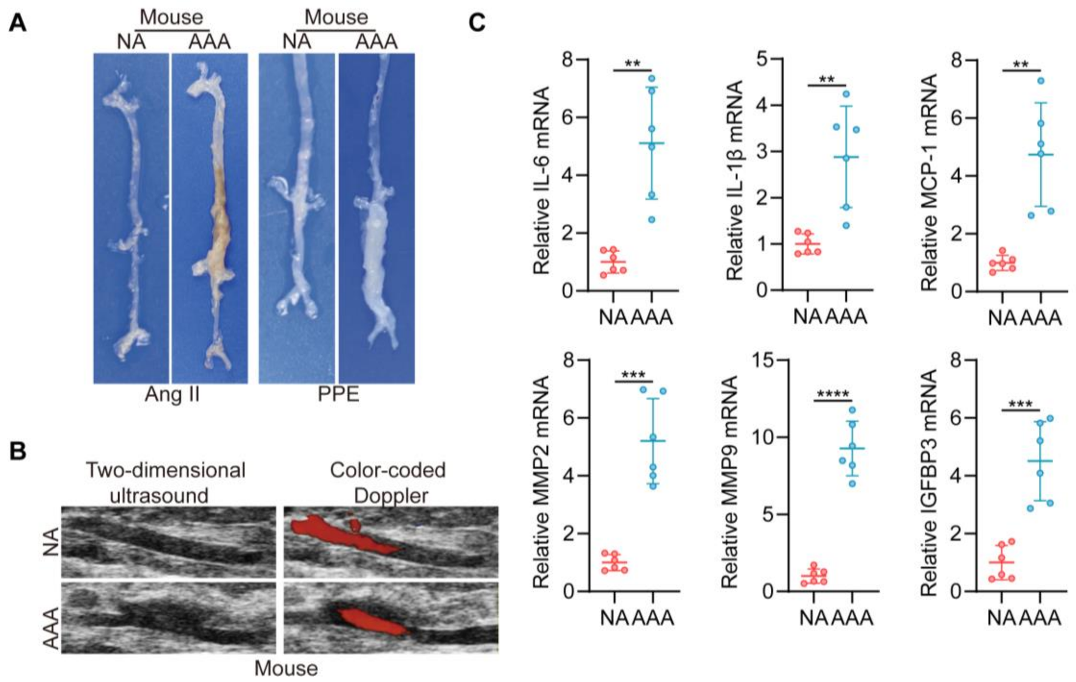

Supplementary Figure 3. Successful establishment of AAA models and significantly increased SASP expression in elastase-induced AAA samples. (A) Representative photographs showing the macroscopic features of aortic aneurysms at 28d after Ang II infusion in male ApoE$^{-/-}$ mice or at 2wk after elastase treatment in male C57BL/6J mice. **(B)** Two-dimensional color-coded ultrasound imaging of aortic aneurysms at 28d after Ang II infusion in male ApoE$^{-/-}$ mice. **(C)** PCR analysis of SASP in aortas from elastase-treated C57BL/6J mice and saline-treated C57BL/6J mice (n = 6/group). RNA levels were normalized to GAPDH. Parametric unpaired t test for Supplementary Fig. 3C. **P < 0.01, ***P < 0.001, ****P < 0.0001.

© 2022. Xie J et al. Published online at http://www.aginganddisease.org/EN/10.14336/AD.2023.0215

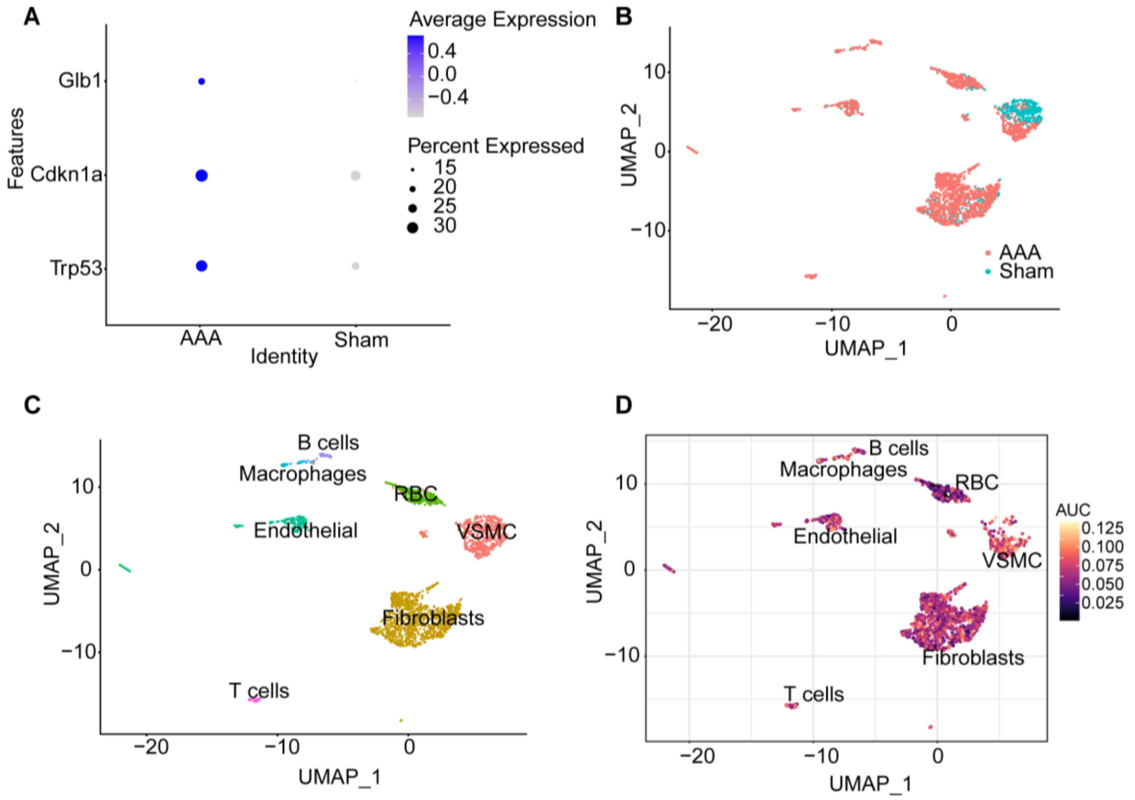

Supplementary Figure 4. Single-cell RNA-sequencing analysis suggests VSMC as the main cell type of SIPS in AAA model. (A) The expression levels of cellular senescence biomarkers, including Glb1, Cdkn1a and Trp53, in Ang II-induced AAA models analyzed from single-cell RNA sequencing data. **(B)** Corresponding UMAP visualisation coloured by sample identity (Sham, AAA). **(C)** UMAP plot of abdominal aortic cell clusters following Ang II infusion representing all of the aortic cell types. Cells in each cluster were identified based on the expression of known cell markers. Colors represent different cell types. **(D)** The AUCell package calculation identifying cells with active senescence-associated gene sets in single-cell RNA-seq data.

© 2022. Xie J et al. Published online at http://www.aginganddisease.org/EN/10.14336/AD.2023.0215

SUPPLEMENTARY DATA

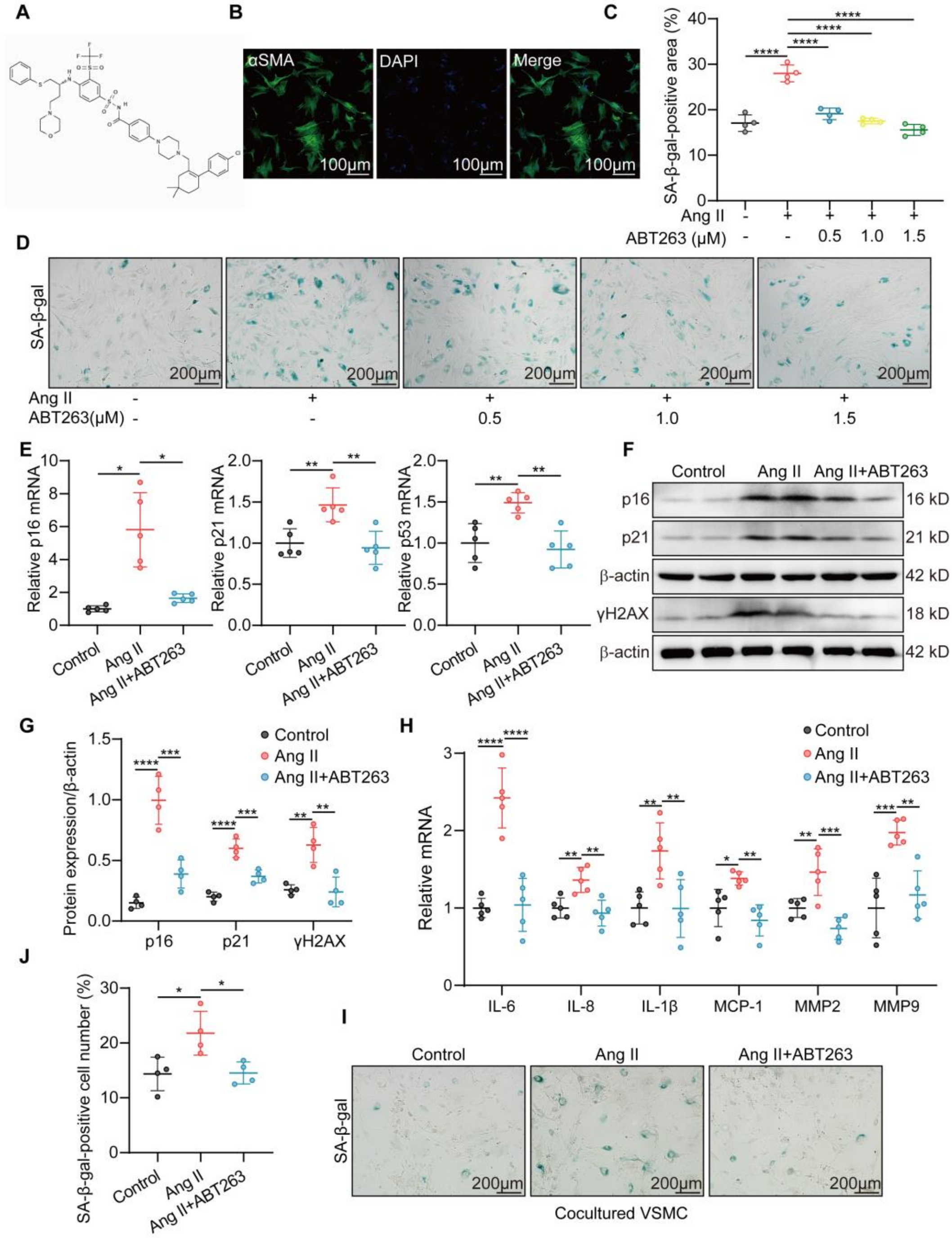

Supplementary Figure 5. ABT263 eliminates stress-induced premature senescent VSMCs and attenuates SASP. (A) Chemical structure of ABT263. **(B)** Immunofluorescence staining for DAPI (blue) and αSMA (green) in mouse primary VSMCs (scale bars = 100 µm). **(C-D)** SA-β-gal staining in control VSMCs (Control), Ang II-induced premature senescent VSMCs treated with vehicle (Ang II) or ABT263 (Ang II+ABT263) at various concentrations (0.5 µM, 1.0 µM and 1.5 µM) (scale bars = 200 µm; n = 4/group). (**E**) RT-PCR assessment of p16, p21 and p53 in the indicated experimental groups (n = 5/group). RNA levels were normalized to GAPDH. (**F-G**) Western blotting and densitometric analysis of p16, γH2AX and p21 in the indicated groups (n = 4/group). **(H)** RT-PCR assessment of IL-6, IL-8, IL-1β, MCP-1, MMP2 and MMP9 in the control group, Ang II group and Ang II+ABT263 group (n = 5/group). RNA levels were normalized to GAPDH. **(I-J)** SA-β-gal staining in VSMCs cocultured with VSMCs from Control group, Ang II group and Ang II+ABT263 group (scale bars = 200 µm; n = 4/group). One-way ANOVA with a post Bonferroni's multiple comparisons test for Supplementary Fig. 5C-J. *P < 0.05, **P < 0.01, ***P < 0.001, ****P < 0.0001.

© 2022. Xie J et al. Published online at http://www.aginganddisease.org/EN/10.14336/AD.2023.0215

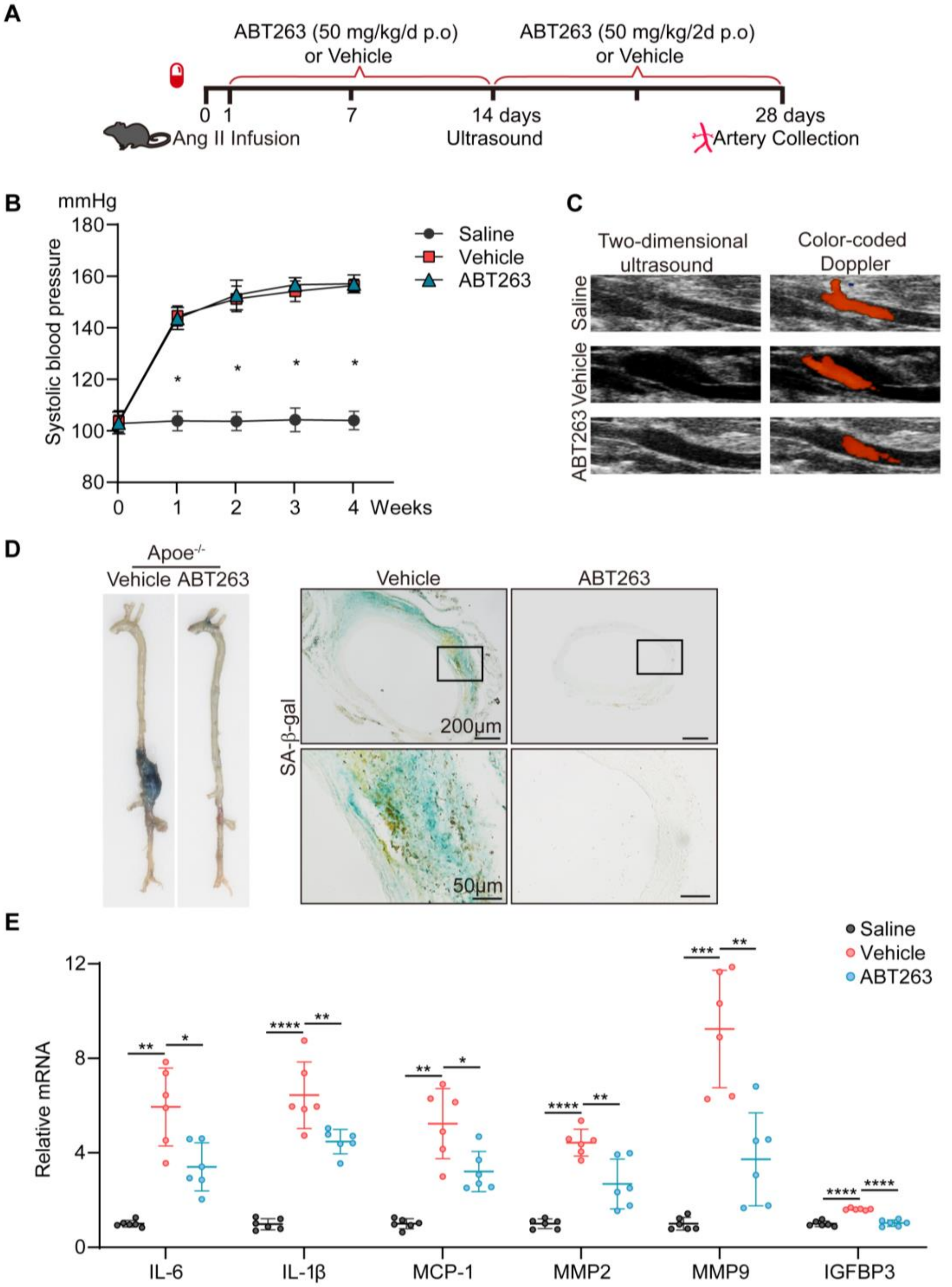

Supplementary Figure 6. ABT263 suppresses cellular senescence in Ang II-perfused AAA models. (A) Mice were either subjected to Ang II infusion or received saline infusion (Saline). Ang II-infused mice were randomly assigned to Vehicle or ABT263 groups and provided with vehicle or ABT263 daily from 1 day after Ang II infusion for 14 days and every other day in the last two weeks. **(B)** Systolic blood pressure at baseline and the indicated weeks after Ang II or saline treatment (n = 10/group). *P < 0.05 Vehicle vs. Saline at each time point. **(C)** Two-dimensional color-coded ultrasound imaging of aortic aneurysms after 14 d of Ang II treatment. **(D)** Representative images of SA-β-gal assay in vehicle-treated and ABT263-treated groups. **(E)** PCR analysis of SASP factors in aorta samples from Saline group, Vehicle group and ABT263 group (n = 6/group). RNA levels were normalized to GAPDH. One-way ANOVA with a post Bonferroni's multiple comparisons test for Supplementary Fig. 6B and 6E. *P < 0.05, **P < 0.01, ***P < 0.001, ****P < 0.0001.

© 2022. Xie J et al. Published online at http://www.aginganddisease.org/EN/10.14336/AD.2023.0215

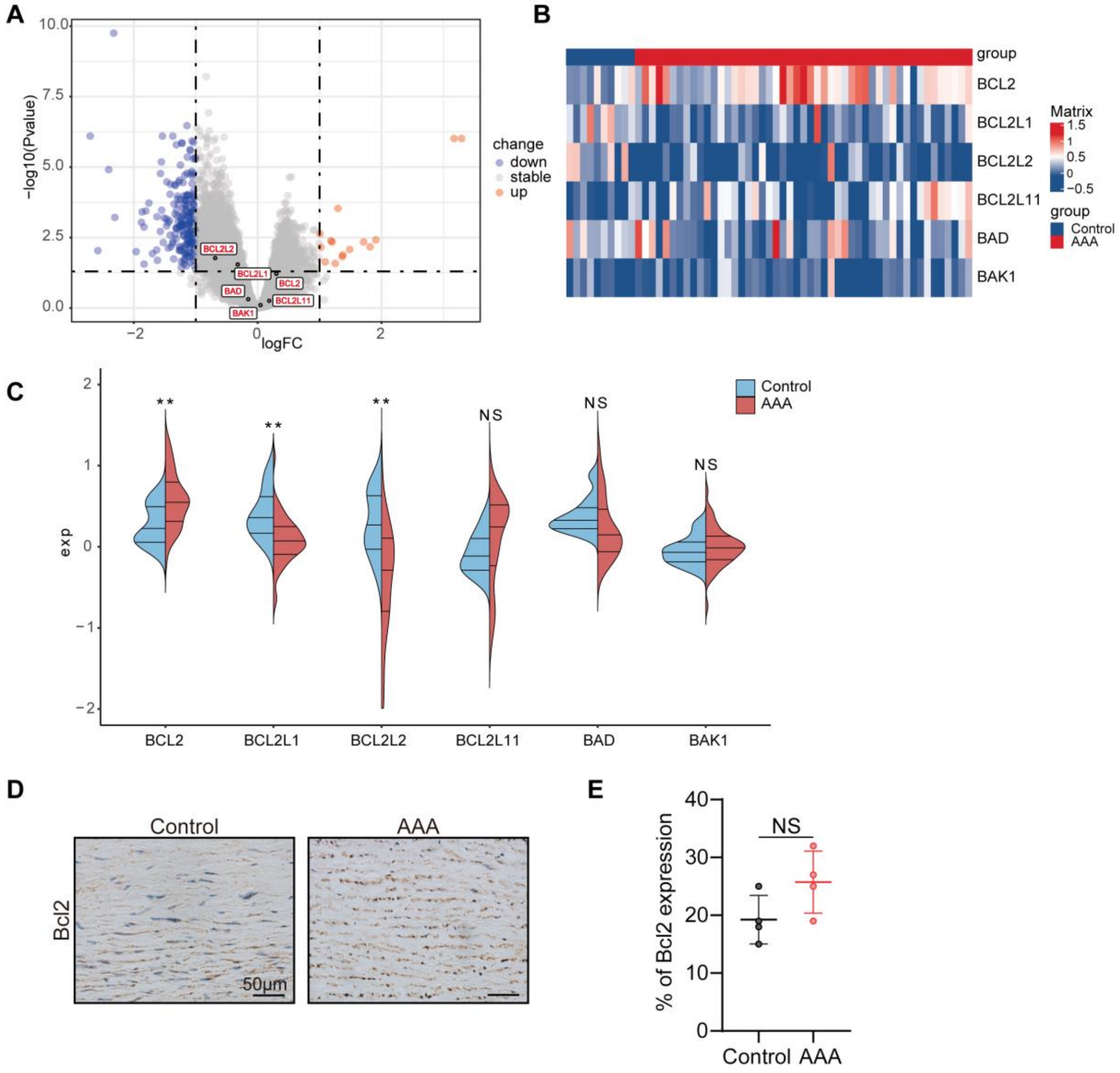

Supplementary Figure 7. The expression levels of Bcl-2 family genes in human AAA tissues and normal aortic tissues. (A) Volcano plot of genes expressed in human AAA tissues (n = 49) and control aortic tissues (n = 10). The down-regulated genes expressed in AAA samples compared with control aortic tissues were plotted in blue, up-regulated genes were in red, and nonsignificant genes were in gray. A P value < 0.05 and absolute fold change > 2 were set as the thresholds for significantly differential expression. **(B)** Heatmap of Bcl-2 family genes in human AAA tissues and normal aortic tissues. **(C)** Violin plot of standardized expression of Bcl-2 family genes according to RNA-sequencing data. **(D-E)** Representative immunohistochemical staining for Bcl2 in human AAA tissues and adjacent aortic tissues (scale bars = 50 μm; n = 4/group). Parametric paired t test for Supplementary Fig. 7E. NS means no significance. **P < 0.01 vs. Control group.

© 2022. Xie J et al. Published online at http://www.aginganddisease.org/EN/10.14336/AD.2023.0215

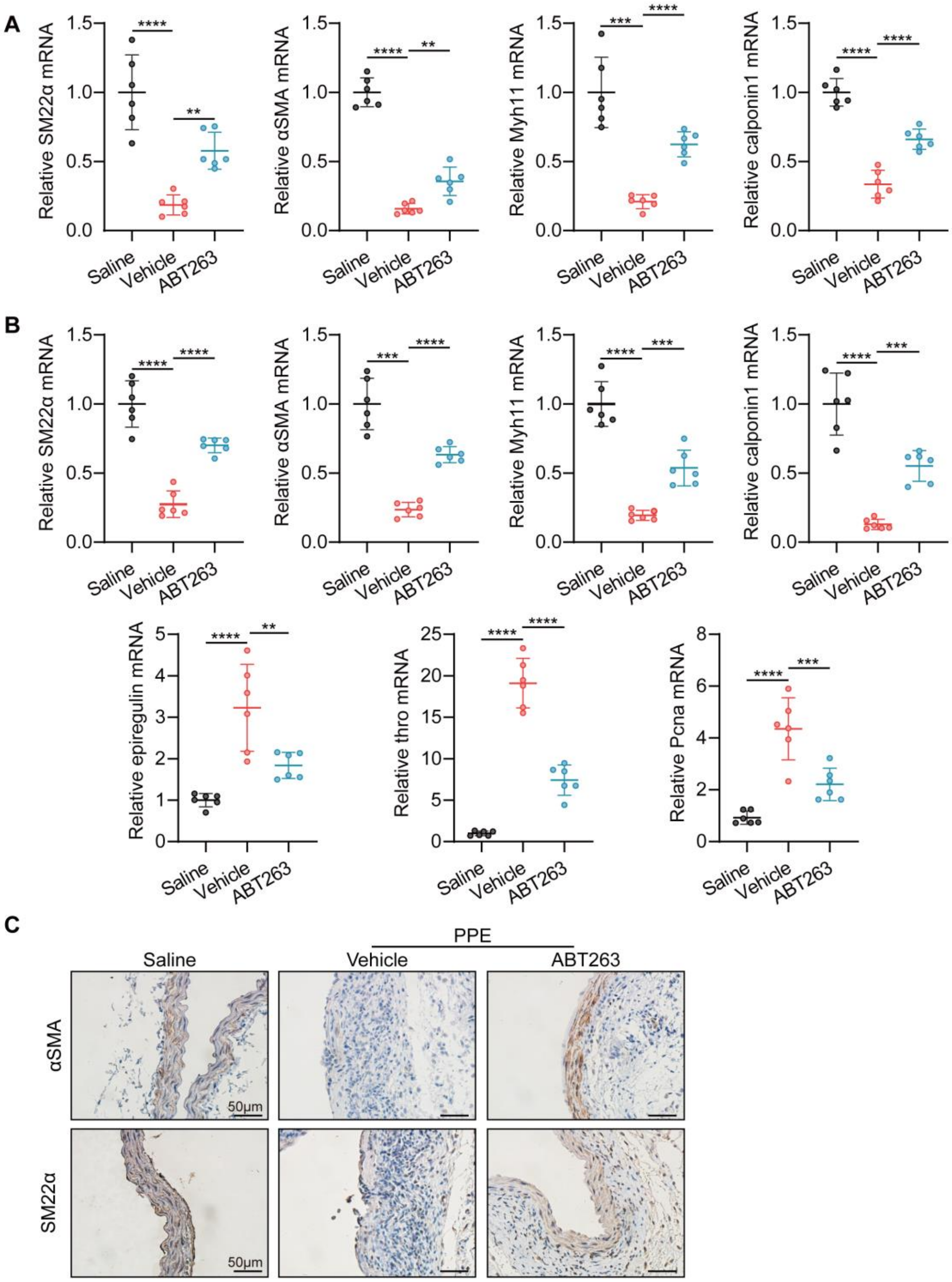

Supplementary Figure 8. ABT263 attenuates VSMC phenotypic switching in AAA models. (A) PCR analysis of VSMC contractile genes in suprarenal aortas in ApoE$^{-/-}$ mice treated with saline (Saline) and in Ang II-treated ApoE$^{-/-}$ mice provided with vehicle (Vehicle) or ABT263 (ABT263) (n = 6/group). RNA levels were normalized to GAPDH. **(B)** PCR analysis of VSMC contractile genes and synthetic genes in infrarenal aortas in C57BL/6J mice treated with saline (Saline) and in elastase-treated C57BL/6J mice provided with vehicle (Vehicle) or ABT263 (ABT263) (n = 6/group). RNA levels were normalized to GAPDH. **(C)** Representative immunohistochemical staining for αSMA and SM22α in infrarenal aortas from saline-treated mice and elastase-treated mice with vehicle or ABT263 administration (scale bars = 50 μm). One-way ANOVA with a post Bonferroni's multiple comparisons test for Supplementary Fig. 8A-B. **P < 0.01, ***P < 0.001, ****P < 0.0001.

SUPPLEMENTARY DATA

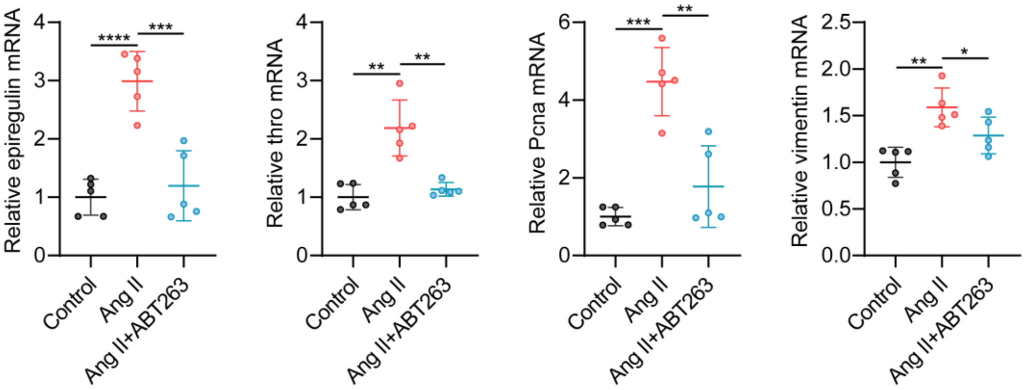

Supplementary Figure 9. ABT263 attenuates expression of VSMC synthetic markers via SASP reduction. mRNA expression levels of VSMC synthetic markers in VSMCs from Control group, Ang II group and Ang II+ABT263 group (n = 5/group). RNA levels were normalized to GAPDH. One-way ANOVA with a post Bonferroni's multiple comparisons test for Supplementary Fig. 9. *P < 0.05, **P < 0.01, ***P < 0.001, ****P < 0.0001.

SUPPLEMENTARY DATA

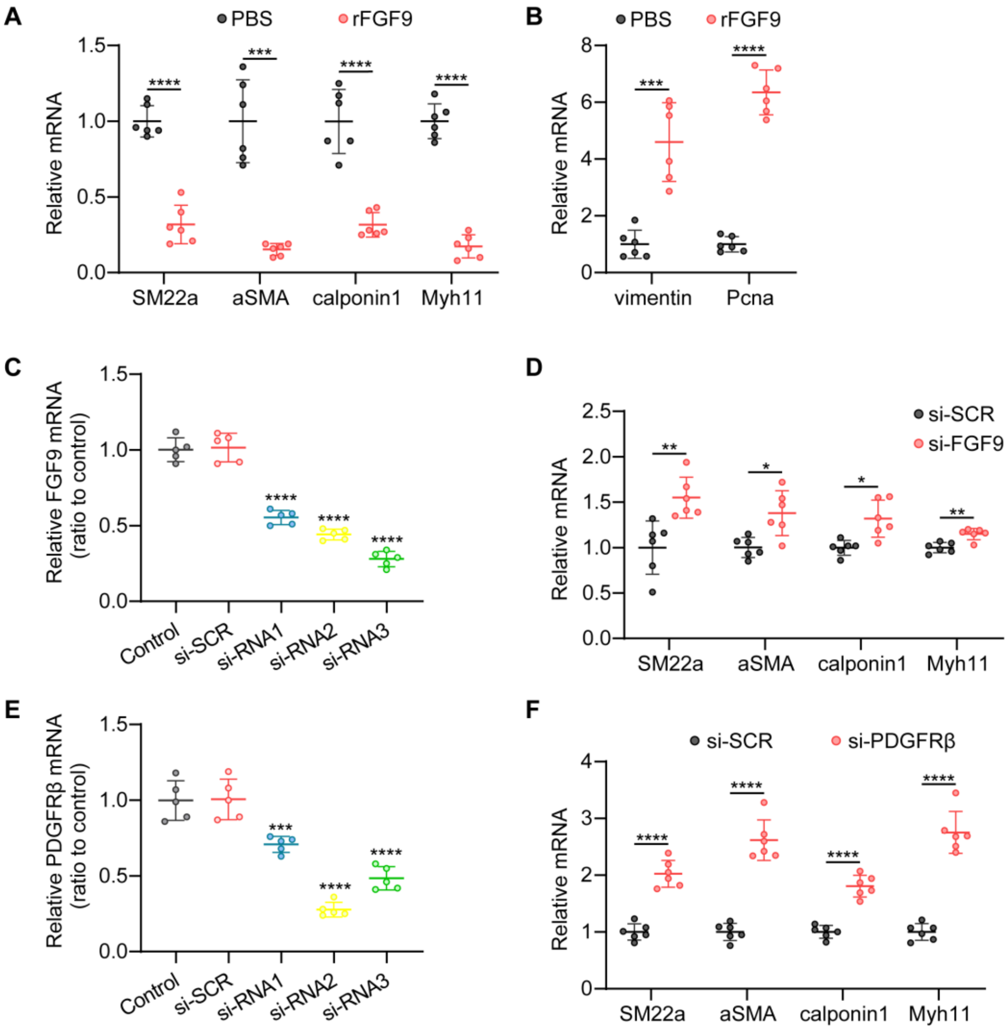

Supplementary Figure 10. FGF9 mediates VSMC phenotypic switching by increasing the expression of PDGFRβ. (A) Expression of VSMC contractile markers measured by RT-PCR in VSMCs incubated with PBS (PBS) or rFGF9 (rFGF9) (n = 6/group). RNA levels were normalized to GAPDH. **(B)** mRNA expression levels of VSMC synthetic markers in VSMCs from PBS group or rFGF9 group (n = 6/group). RNA levels were normalized to GAPDH. **(C)** mRNA expression level of FGF9 in VSMCs incubated with three different FGF9-targeting siRNAs (n = 5/group). RNA levels were normalized to GAPDH. ****P < 0.0001 vs. Control group. **(D)** mRNA levels of VSMC phenotypic switch markers in VSMCs cocultured with stress-induced premature senescent VSMCs treated with or without FGF9 knockdown (n = 6/group). RNA levels were normalized to GAPDH. **(E)** PDGFRβ mRNA level in VSMCs incubated with three different PDGFRβ-targeting siRNAs (n = 5/group). RNA levels were normalized to GAPDH. ****P < 0.0001 vs. Control group. **(F)** RT-PCR assessment of VSMC contractile markers in rFGF9-treated VSMCs with or without PDGFRβ knockdown (n = 6/group). RNA levels were normalized to GAPDH. One-way ANOVA with a post Bonferroni's multiple comparisons test for Supplementary Fig. 10C and 10E, and parametric unpaired t test for Supplementary Fig. 10A-B, 10D and 10F. *P < 0.05, **P < 0.01, ***P < 0.001, ****P < 0.0001.

© 2022. Shoeb F et al. Published online at http://www.aginganddisease.org/EN/10.14336/AD.2022.0830
